# Umbrella Review of Systematic Reviews and Meta-Analyses on Consumption of Different Food Groups and Risk of Type 2 Diabetes Mellitus and Metabolic Syndrome

**DOI:** 10.1016/j.tjnut.2025.03.021

**Published:** 2025-03-22

**Authors:** Rivana Lambani Banjarnahor, Elaheh Javadi Arjmand, Anindita Tasnim Onni, Lise M Thomassen, Matteo Perillo, Rajiv Balakrishna, Ida Sofie Karlsen Sletten, Antonello Lorenzini, Pierluigi Plastina, Lars T Fadnes

**Affiliations:** 1 Department of Global Public Health and Primary Care, University of Bergen, Bergen, Norway; 2 Department of Pharmacy, Health and Nutrition Sciences, University of Calabria, Arcavacata di Rende, Italy; 3 Department of Addiction Medicine, Haukeland University Hospital, Bergen, Norway; 4 Department of Biomedical and Neuromotor Sciences, University of Bologna, Bologna, Italy; 5 The Medical Library, University of Bergen, Bergen, Norway; 6 Biostructures and Biosystems National Institute (INBB), Roma, Italy

**Keywords:** food, nutrition, diet, food groups, mortality, type 2 diabetes, metabolic syndrome, noncommunicable diseases, meta-analysis, systematic review

## Abstract

Type 2 diabetes is a major contributor to the burden of chronic diseases globally. Most cases of type 2 diabetes are preventable through healthy lifestyle modifications in diet and physical activity. This systematic umbrella review presents a comprehensive overview of the evidence about the associations between risk of type 2 diabetes and metabolic syndrome with 13 food groups, including refined and whole grains, fruits, vegetables, nuts, legumes, fish and fish products, eggs, dairy/milk, sugar-sweetened beverages, processed meat, and unprocessed red and white meat. We present these relationships in per-serving and with high-versus-low comparisons. After doing a systematic search in MEDLINE, Embase, Web of Science, and Epistemonikos (registered with PROSPERO: CRD42024547606), we screened 5074 references published until May 15, 2024, and included 67 articles. This included 46 meta-analyses on risk of type 2 diabetes with half a million participants, 17 meta-analyses on risk of metabolic syndrome, and 4 meta-analyses on risk of diabetes-related mortality. Based on quality assessments using AMSTAR-2, 25 of the 67 studies were classified as high-quality studies, 8 as moderate, 12 as low, and 22 as critically low quality. Our results showed that a high intake of whole grains was associated with a lower risk of type 2 diabetes (metaevidence: moderate) and metabolic syndrome (metaevidence: low), with a similar tendency also for a high intake of fruits and vegetables (metaevidence: moderate). In contrast, the high intakes of processed meat (metaevidence: high), red meat (metaevidence: moderate), and sugar-sweetened beverages (metaevidence: moderate) were associated with a higher risk of type 2 diabetes. For the other food groups, the associations were generally neutral and not statistically significant. The heterogeneity was high for most food groups except fruits, indicating potential differences within each of the food groups in association with type 2 diabetes.

## Introduction

Noncommunicable diseases such as cardiovascular disease, cancer, and metabolic disorders are among the leading contributors to global mortality [1,2]. Although diabetes was the 10th leading cause of death, it is a major risk factor for ischemic heart disease [1,3]. More than half a billion people worldwide have diabetes, with ∼24 million new cases in 2021 [2,4]. This number is projected to increase to 783 million people by 2045 [5]. Although these numbers include all types of diabetes, type 2 diabetes contributes to the majority of these and has a strong association with obesity and an unhealthy lifestyle [6]. Type 2 diabetes is generally linked with insulin resistance and hyperinsulinemia [7]. However, type 2 diabetes is also reflected in a combination of traits defined as metabolic syndrome [8]. The criteria for metabolic syndrome can differ but involve hypertension, hyperglycemia, raised levels of triglycerides, low levels of high-density lipoprotein, and large waist circumference [9,10]. Evidence indicates that most type 2 diabetes cases can be prevented or delayed with sufficient lifestyle modifications [11].

Dietary assessments and interventions are essential components of lifestyle modifications aimed at the prevention and management of type 2 diabetes [12,13]. Understanding how different food groups contribute to the risk of type 2 diabetes mellitus and metabolic disease is crucial not only for informing public health recommendations and dietary guidelines but also in clinical contexts among people at risk. Despite substantial research on individual dietary components and their effects on health outcomes, there is to our knowledge no systematic umbrella review that integrates recent evidence on the relationship between consumption of various food groups and risk of type 2 diabetes mellitus and metabolic syndrome.

By focusing on high-versus-low and dose–response relationships, this systematic umbrella review seeks to offer a comprehensive assessment of eating categorized through the intake of various food groups and its association with risk of type 2 diabetes mellitus and metabolic syndrome.

## Methods

To summarize the evidence from meta-analyses and systematic reviews regarding the consumption of various food groups and their associated health outcomes on type 2 diabetes mellitus and metabolic syndrome, we utilized an umbrella review framework. The food groups included edible refined and whole grains, fruits, vegetables, nuts, legumes, fish and fish products, eggs, milk and dairy products, meat and meat products (red and white unprocessed and processed meats), sugar-sweetened beverages, and added sugars. The study protocol has been registered with PROSPERO (CRD42024547606).

### Eligibility criteria

We evaluated meta-analyses and systematic reviews that included analyses from cohort studies and randomized controlled trials regarding the consumption of various food groups [edible grains (both refined and whole), fruits, vegetables, nuts, legumes, fish and fish products, eggs, milk and dairy products, meat and meat products (including red, processed, and white meats), sugar-sweetened beverages, and added sugars] and their associations with risk of type 2 diabetes mellitus and metabolic syndrome. Detailed inclusion and exclusion criteria are outlined in Text box 1. Studies with cross-sectional designs or those providing regional estimates not generalizable to broader populations were excluded. No restrictions were applied to publication date or publication status, although only articles published in English were considered. We conducted outcome measures with risk of hazard ratios (HRs), odds ratios, and absolute risk differences.

Text box 1 **Inclusion and exclusion criteria**

**Table:** 

**Inclusion criteria:**
• Study types: Meta-analysis and systematic reviews presenting analyses from longitudinal observation studies (e.g., cohorts, nested case–control) and trials
• Exposure: Consumption of 14 food groups (edible grains including refined and whole grains, fruit, vegetables, nuts, legumes/pulses, fish and fish products, eggs, milk/dairy, meat and meat products including red, processed, white meat, sugar-sweetened beverages, and added sugars)
• Comparators: High vs. low consumption, per-serving and dose–response relationship between exposure and outcomes
• Outcome: Incidence and mortality of type 2 diabetes mellitus
• Secondary outcomes: Incidence and mortality of metabolic syndrome (as a combined syndrome)
• Language: English
• Publication status: Published articles indexed in MEDLINE, Embase, Cochrane, and Epistemonikos from inception to May 15, 2024
**Exclusion criteria:**
• Umbrella reviews
• Animal studies
• Studies on only specific regions
• Studies only reporting on macronutrients or micronutrients
• Large proportion of the study population has type 1 diabetes

Alt-text: Text box 1

### Information sources

**FIGURE 1:**
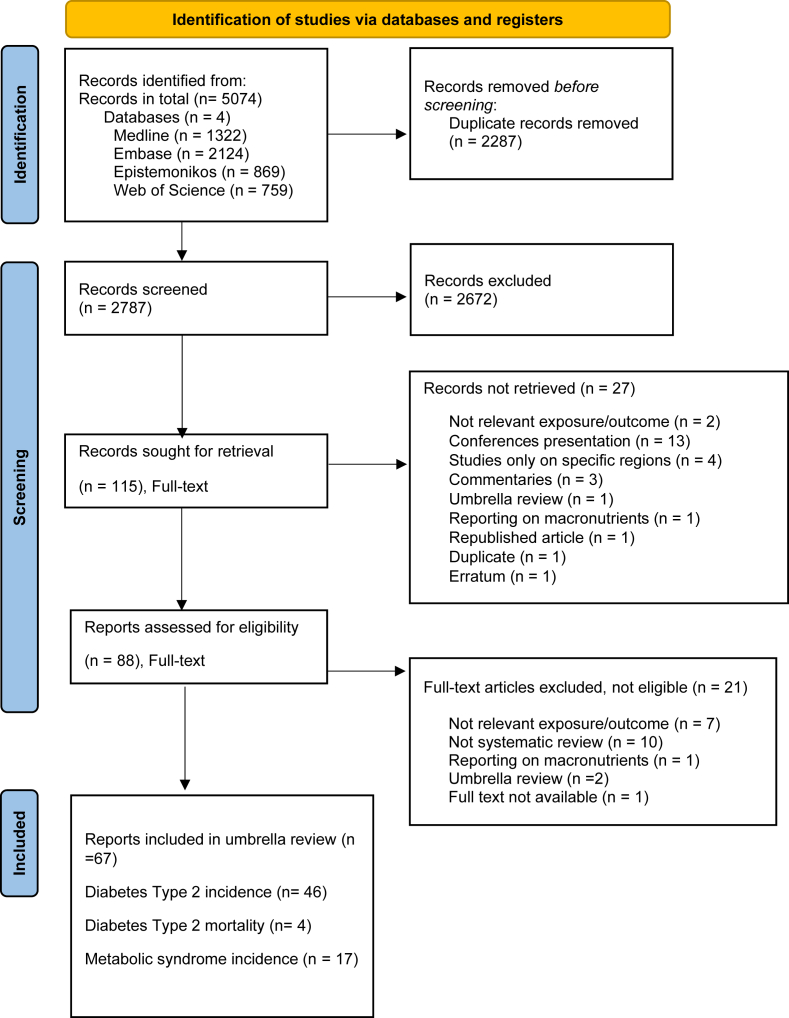
Preferred Reporting Items for Systematic Reviews and Meta-Analyses (PRISMA) flowchart.

In collaboration with an academic librarian, a total of 5074 records were retrieved from the databases MEDLINE, Embase, Epistemonikos, and Web of Search. The search covered the period from database inception to 15 May, 2024. After automatic deduplication using EndNote, 2787 records remained (Figure 1 and Supplemental Text 1 for the full search queries). No restrictions were applied to the publication date. The search was reviewed by another academic librarian. This systematic review was conducted in accordance with the Preferred Reporting Items for Systematic Reviews and Meta-Analyses (PRISMA) guidelines [14].

### Search

The search included the following terms (≥1 element of each point):

1. Edible grains (including refined grains), whole grains, fruits, vegetables, nuts, legumes, pulses, fish products, eggs, milk, dairy, meat and meat products (including unprocessed red and white meat, processed meat), sugar-sweetened beverages, or added sugars
2. Intake, consumption, eat, or diet
3. Systematic review or meta-analysis
4. Type 2 diabetes or metabolic syndrome

The search included relevant free text words as well as relevant MeSH/Emtree subject headings (Supplemental Text 1). The references were managed using EndNote 21.

### Study selection

After performing a deduplication of the retrieved studies, 2 authors (R.L.B. and E.J.A.) independently screened the references, with disagreement resolved by consensus of another reviewer (A.T.O.) based on the inclusion and exclusion criteria. One author conducted screening manually through all articles; screening involved reviewing titles and abstracts, followed by a full-text assessment of articles deemed potentially relevant. Another author did it using ASReview, v.1.6.3rc0 [15]. ASReview, developed at Utrecht University, is an active-learning–powered tool that learns from the assessments performed by the reviewer and iteratively provides them with an updated list of the records more likely to be relevant [16]. Eventually, 122 were considered potentially eligible.

### Data collection process and data items

Data considered relevant was extracted into a Microsoft Excel table. The information gathered included study design and aims, methods (including inclusion and exclusion criteria), and population(s)/participants (including sample size). The data focused on food groups as interventions or exposures, including specifications, comparators, type 2 diabetes, and metabolic syndrome as outcomes. Additional details included the search period, year of publication, funding sources, conflicts of interest reported, first author, and characteristics, such as the number of studies, heterogeneity, and findings (e.g., high vs. low intake, dose–response relationships, or findings categorized by serving size). Three authors (R.L.B., E.J.A., and A.T.O.) extracted the data independently and double-checked each other's extractions. Details of the extractions are provided in Supplemental Table 1.

### Risk of bias and quality of metaevidence

Risk of bias was assessed using the AMSTAR-2 tool [17]. Reviews were assessed by 3 authors independently (R.L.B., E.J.A., and L.M.T.), and they were rated as high, moderate, or low quality based on this tool (e.g., AMSTAR-2: high). Three authors (L.T.F., A.L., and P.P.) checked these ratings, and any disagreement was discussed and resolved by consensus. Details of these assessments are provided in Supplemental Table 2.

The quality of the metaevidence for the association between each food group and outcome was assessed using the NutriGrade tool [18]. The NutriGrade scoring system for cohort studies involves 8 items:

1. Risk of bias (mean score for included studies in the meta-analysis: 0–2 points)
2. Precision (0–1 point)
3. Heterogeneity (0–1 point)
4. Directness (0–1 point)
5. Publication bias (0–1 point)
6. Funding bias (0–1 point)
7. Effect size (0–2 point)
8. Dose–response analysis (0–1 point)

The overall score was categorized as high (≥8 points, meaning further research probably will not change the confidence in the effect estimate), moderate (6–7.9, further research could add evidence to the confidence and may change the effect estimate), low (4–5.9, further research will provide important evidence on the confidence and likely change the effect estimate), or very low (0–3.9, very limited certainty for the effect estimate) [18].

### Analysis

We have presented the extracted data from the included studies and their AMSTAR-2 scores in tables. These data were summarized in figures visualizing the associations between food groups and each outcome (incidence of type 2 diabetes, incidence of metabolic syndrome, and diabetes-related mortality) both for low versus high comparison and dose–response. We have presented a table for each combination of food group and comparison (high vs. low, per-serving/linear dose–response, and nonlinear dose–response). Each table includes the following information: first author, search year, number of primary studies, number of participants, number of observed events, estimated association with each outcome, heterogeneity for high-versus-low and linear dose–response, intake range for the nonlinear dose–response analysis, and AMSTAR-2 score. These tables are presented in the Supplemental Material. For per-serving comparisons, we presented a converted HR to facilitate comparison across studies [HR_standardized_ = HR_reported_ (standardized dose/reported dose), with analogous calculations for the confidence intervals]. The standardized dose–response was established based on the subsequent serving sizes: added sugars: 10 g/d; dairy products: 200 g/d (in milk equivalents); eggs: 50 g/d; fish: 100 g/d; fruits: 80 g/d; legumes: 50 g/d; nuts: 28 g/d; processed meat: 50 g/d; unprocessed red meat: 100 g/d; refined grains: 30 g/d (product weight/fresh weight); sugar-sweetened beverages: 250 g/d or 250 mL/d; vegetables: 100 g/d; white meat: 100 g/d; and whole grains: 30 g/d (product weight/fresh weight). We used standard recommended conversions for studies that reported consumption only as serving size without providing quantitative amounts. The same data were also utilized to create forest plots that visually summarize the relationships found in the meta-analysis for high-versus-low and per-serving comparisons. The meta-analysis with the same exposure and outcome can present overlapping cohort studies. To present a meta-analysis including most of the available cohorts, we used a decision tree to identify the most recent and comprehensive study for the association between each food group and each outcome. This was done in the following steps: First, looking at the quality of the evidence, the reviews with low AMSTAR-2 ratings were ruled out unless there was no study without a better-assessed quality. Second, we selected the reviews with the most included studies, participants, and reported events. Among these candidates, we selected the most recent review. These studies were then rated using the NutriGrade scoring system (Supplemental Table 3). We included 2 forest plots and a table that details the findings and features of the most recent and comprehensive meta-analyses (1 for each combination of exposure and comparison). All meta-analyses, including those that were not deemed the most recent or comprehensive, are presented in the Supplemental Materials, including Supplemental Tables 4–6 and Supplemental Figures 1–34. Data preprocessing, result conversion, and the generation of tables and plots were performed using R, version 4.4.1, developed by the R Core Team.

## Results

After screening the full text, 67 articles were included in this systematic umbrella review [including 46 articles with meta-analyses reporting the association of various food groups with incidence of type 2 diabetes, 4 articles on the mortality of type 2 diabetes, and 17 articles reporting on the incidence of metabolic syndrome (Figure 1)] [[19], [20], [21], [22], [23], [24], [25], [26], [27], [28], [29], [30], [31], [32], [33], [34], [35], [36], [37], [38], [39], [40], [41], [42], [43], [44], [45], [46], [47], [48], [49], [50], [51], [52], [53], [54], [55], [56], [57], [58], [59], [60], [61], [62], [63], [64], [65], [66], [67], [68], [69], [70], [71], [72], [73], [74], [75], [76], [77], [78], [79], [80], [81], [82], [83], [84], [85]]. Twenty-one articles were excluded because they either reported irrelevant outcomes, considered macronutrients as exposures, were not systematic reviews of the literature, or were an umbrella review [[86], [87], [88], [89], [90], [91], [92], [93], [94], [95], [96], [97], [98], [99], [100], [101], [102], [103], [104], [105], [106]]; they are reported in Supplemental Table 1.

### Type 2 diabetes incidence

For type 2 diabetes incidence, the most comprehensive and recent studies were conducted between 2017 and 2021 (Table 1) [32,34,40,62,63,65,66,77,80]. For most food groups, the combined data included more than half a million participants and >30,000 cases [32,62,63,65,66,68]. However, for some food groups, the number of participants and cases was lower (eggs, nuts, whole grains for both comparisons, and legumes for high vs. low) [34,65,71,77]. The heterogeneity for most of the studies was higher than 0.5, except for fruits (both high vs. low and per serving) and white meat (per serving) [65,77]. All the articles included cohort studies, but only 1 article also included case–control studies [65]. There were no randomized controlled trials involved in the articles. The median number of the included cohort studies per meta-analysis was 13.5 (ranging from 7 to 23).

**TABLE 1:** Overview of the most comprehensive/up-to-date meta-analyses on associations between food groups and incidence of type 2 diabetes with details on first author, search year, reference number, exposure, number of studies, participants, events, results, and NutriGrade score.

Food group	Author (year)	Study quality^1^	Comparison	Studies/participants/events	Results	Certainty of evidence^b^
Dairy	Schwingshackl et al. (2017) [65]	Moderate	HL	21/566867/44474	0.91 (0.85, 0.97) (*I*^2^ = 0.63)	Moderate
Dairy	Schwingshackl et al. (2017) [65]	Moderate	PS (200 g)	21/566872/44474	0.97 (0.94, 0.99) (*I*^2^ = 0.74)	Moderate
Eggs	Schwingshackl et al. (2017) [65]	Moderate	HL	13/315358/17629	1.08 (0.95, 1.22) (*I*^2^ = 0.69)	Low
Eggs	Fan et al. (2019) [32]	Low	PS (50 g)	19/374891/20691	1.01 (0.99, 1.03) (*I*^2^ = 0.54)	Moderate
Fish	Fan et al. (2019) [32]	Low	HL	20/698894/43239	1.08 (1.00, 1.18) (*I*^2^ = 0.77)	Moderate
Fish	Fan et al. (2019) [32]	Low	PS (100 g)	8/698894/43239	0.98 (0.85, 1.14) (*I*^2^ = 0.61)	Moderate
Fruit	Halvorsen et al. (2020) [40]	High	HL	20/1532167/81313	0.93 (0.90, 0.97) (*I*^2^ = 0.09)	Moderate
Fruit	Halvorsen et al. (2020) [40]	High	PS (80 g)	19/1496860/80994	0.98 (0.97, 1.00) (*I*^2^ = 0.68)	Moderate
Legumes	Thorisdottir et al. (2022) [71]	High	HL	10/312874/15992	0.90 (0.77, 1.06) (*I*^2^ = 0.88)	Low
Legumes	Schwingshackl et al. (2017) [65]	Moderate	PS (50 g)	10/536860/29223	1.00 (0.92, 1.09) (*I*^2^ = 0.87)	Moderate
Nuts	Schwingshackl et al. (2017) [65]	Moderate	HL	8/313847/27016	0.95 (0.85, 1.05) (*I*^2^ = 0.67)	Low
Nuts	Schwingshackl et al. (2017) [65]	Moderate	PS (28 g)	7/297012/15470	0.89 (0.71, 1.12) (*I*^2^ = 0.77)	Low
Processed meat	Schwingshackl et al. (2017) [65]	Moderate	HL	14/550342/43781	1.27 (1.20, 1.35) (*I*^2^ = 0.55)	High
Processed meat	Shi et al. (2022) [66]	Moderate	PS (50 g)	20/1187449/NA	1.44 (1.27, 1.63) (*I*^2^ = 0.91)	Moderate
Red meat	Zhang et al. (2021) [80]	High	HL	14/674345/50007	1.15 (1.08, 1.23) (*I*^2^ = 0.68)	Moderate
Red meat	Shi et al. (2022) [66]	Moderate	PS (100 g)	23/1650241/NA	1.27 (1.16, 1.39) (*I*^2^ = 0.89)	Moderate
Refined grains	Schwingshackl et al. (2017) [65]	Moderate	HL	14/599772/22559	1.01 (0.92, 1.10) (*I*^2^ = 0.54)	Moderate
Refined grains	Schwingshackl et al. (2017) [65]	Moderate	PS (30 g)	14/599772/22559	1.01 (0.99, 1.03) (*I*^2^ = 0.59)	Moderate
SSBs	Qin et al. (2019) [62]	Moderate	HL	18/1010392/34788	1.27 (1.18, 1.36) (*I*^2^ = 0.80)	Moderate
SSBs	Qin et al. (2019) [62]	Moderate	PS (250 g)	19/1010392/34788	1.19 (1.13, 1.25) (*I*^2^ = 0.82)	Moderate
Vegetables	Halvorsen et al. (2020) [40]	High	HL	17/973814/52959	0.95 (0.88, 1.02) (*I*^2^ = 0.60)	Moderate
Vegetables	Halvorsen et al. (2020) [40]	High	PS (100 g)	15/784014/52333	0.98 (0.97, 1.00) (*I*^2^ = 0.39)	Moderate
White meat	Ramel et al. (2021) [63]	Low	HL	7/1241230/NA	0.98 (0.87, 1.11) (*I*^2^ = 0.82)	Low
White meat	Yang et al. (2019) [77]	Low	PS (100 g)	11/411276/32983	1.08 (1.00, 1.19) (*I*^2^ = 0.00)	Moderate
Whole grains	Ghanbari-Gohari et al. (2021) [34]	Moderate	HL	11/436282/37249	0.79 (0.73, 0.85) (*I*^2^ = 0.77)	Moderate
Whole grains	Ghanbari-Gohari et al. (2021) [34]	Moderate	PS (30 g)	9/436282/37249	0.85 (0.80, 0.92) (*I*^2^ = 0.94)	Low

HL, high versus low; PS, per serving with serving sizes in the parentheses; SSB, sugar-sweetened beverage.

a AMSTAR2 score.

b NutriGrade scoring system for meta-analysis of cohort studies.

**FIGURE 2:**
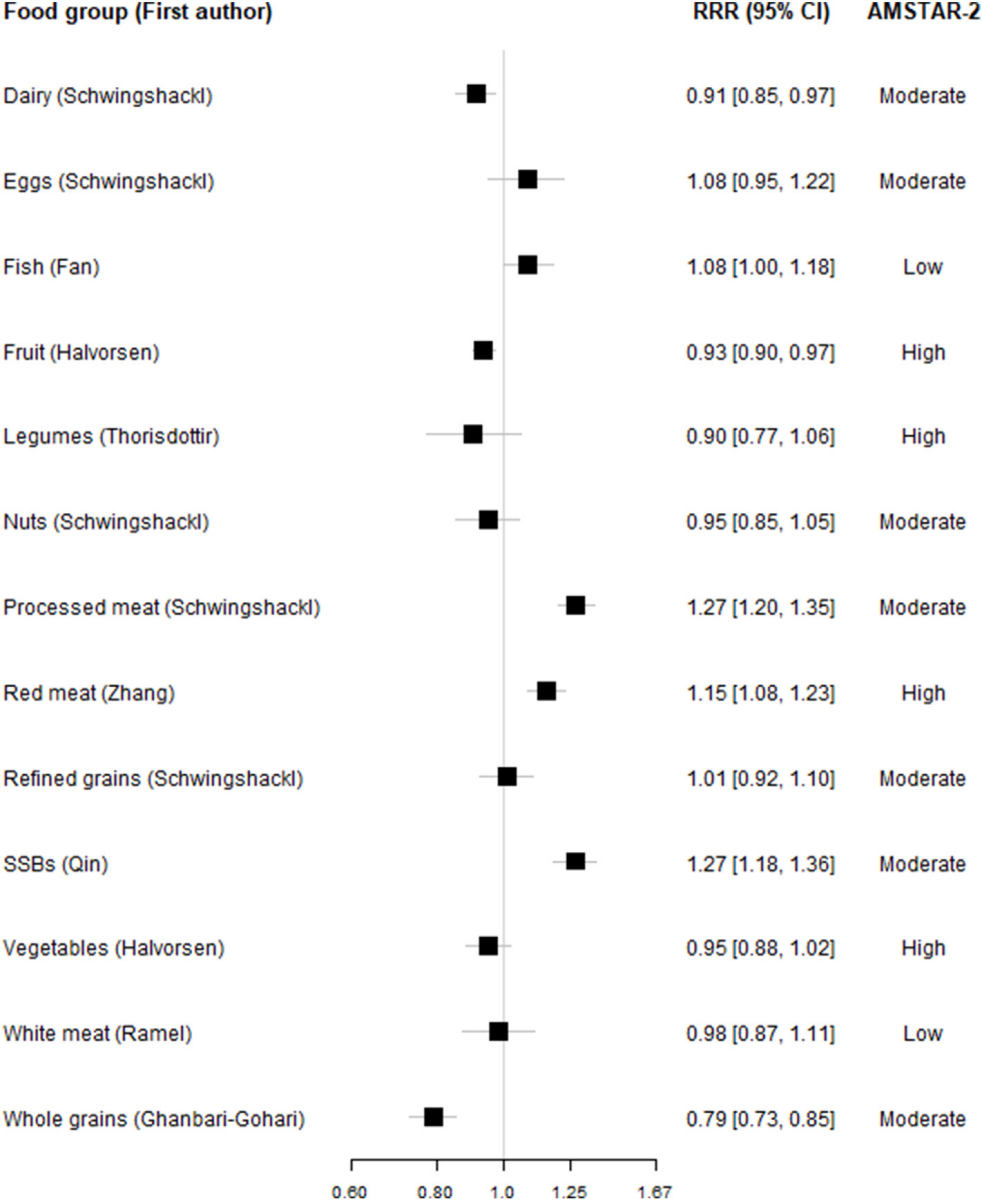
Associations between high-versus-low consumption of each of the food groups and incidence of type 2 diabetes in most comprehensive and up-to-date meta-analyses.

**FIGURE 3:**
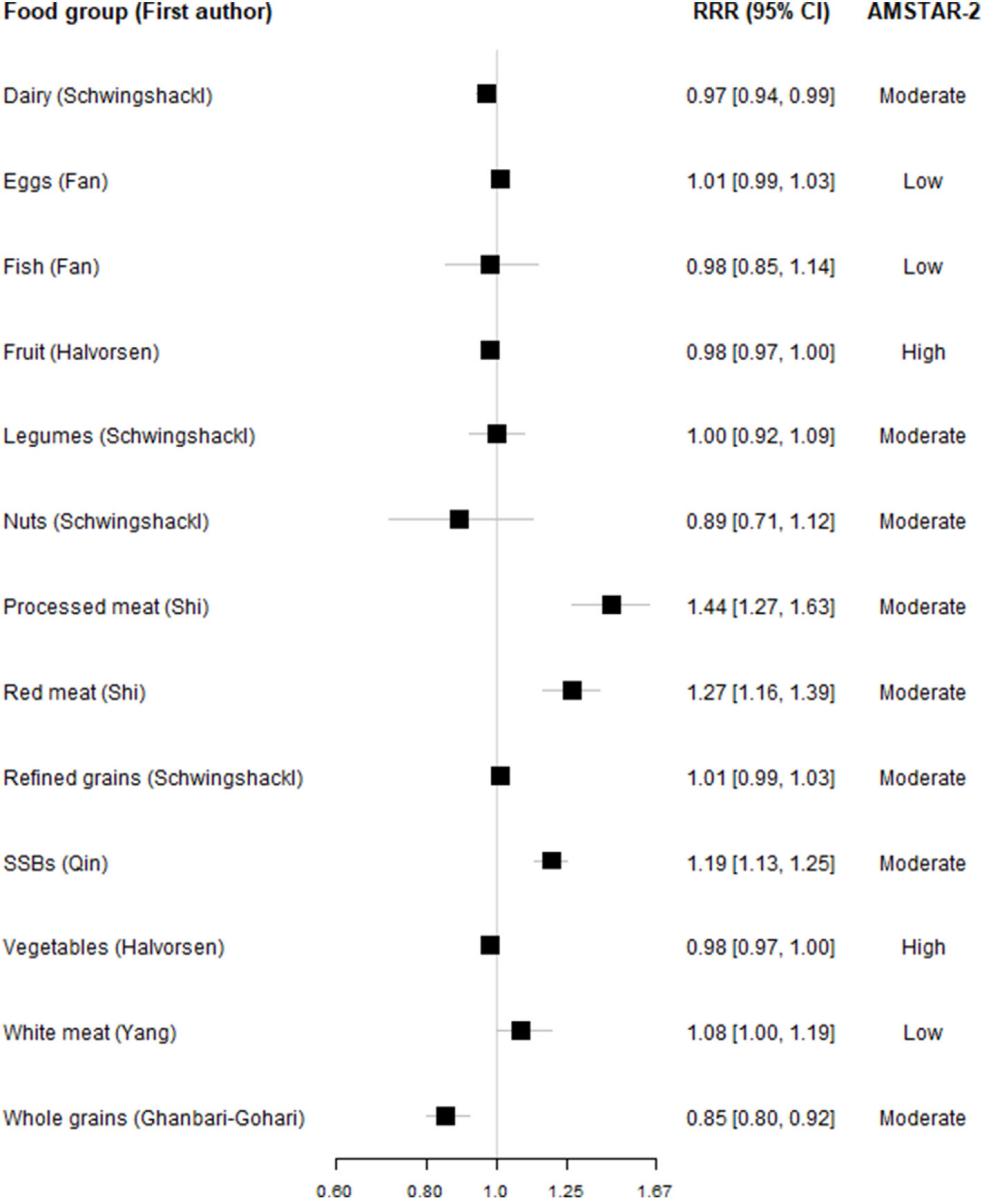
Associations between per-serving comparisons∗ for each of the food groups and incidence of type 2 diabetes in most comprehensive and up-to-date meta-analyses. ∗Serving sizes for each food group (g/d): dairy: 200; eggs: 50; fish: 100; fruits: 80; legumes: 50; nuts: 28; processed meat: 50; red meat: 100; refined grains: 30 (product weight/fresh weight); sugar-sweetened beverages: 250 or 250 mL/d; vegetables: 100; white meat: 100; and whole grains: 30 (product weight/fresh weight).

The meta-analysis on high-versus-low comparisons (Figure 2) and per-serving comparisons (Figure 3) showed that a high intake of whole grains is associated with a lower risk of type 2 diabetes. In contrast, a higher intake of processed meat, red meat, and sugar-sweetened beverages is associated with a higher risk of type 2 diabetes. Higher intake of vegetables, fruits, legumes, nuts, white meat, and dairy products had a nonsignificant association with lower risk of type 2 diabetes. In comparison, a higher intake of fish and eggs had a nonsignificant association with a higher risk of type 2 diabetes. We also identified nonlinear dose–response relationships between intake of food groups and incidence of type 2 diabetes, which was in line with per-serving results.

Among 46 articles focusing on the incidence of type 2 diabetes, 15 were rated as high quality using AMSTAR-2 scores, 6 were rated as moderate, 7 as low, and 18 as critically low quality [17]. The quality criteria that were frequently lacking were reporting funding for included studies, registered protocol, duplicated study selection, duplicated study extractions, and tools for assessing bias and confounding (Supplemental Table 2). The most comprehensive and recent studies were rated as moderate and high, with 3 studies rated as low quality (eggs per serving, fish for both comparisons, and white meat for both comparisons). The results of additional meta-analyses can be found in Supplemental Tables 4–6 and Supplemental Figures 1–26.

### Diabetes mortality

Studies focusing on diabetes-related mortality were conducted from 2016 to 2022 (Supplemental Table 7). The median number of participants was 40,966, and the median number of deaths was 1862. The comparison type mostly reported was per serving, whereas high-versus-low comparison was found for fish and whole grains. The reported heterogeneity (*I*^2^) was generally low except for eggs, nuts, and whole grains (high vs. low comparison). All the meta-analyses included cohort studies, with only 1 study also including case–control studies [42]. The median number of included studies was 4 (range: 2–8).

The meta-analysis on high-versus-low (Supplemental Figure 27) and per-serving comparisons (Supplemental Figure 28) indicated that a high intake of fruits, vegetables, whole grains, and fish is associated with a lower risk of mortality for people with diabetes. In contrast, a higher intake of eggs was associated with a higher risk of mortality for people with diabetes. Higher intake of nuts had a tendency toward lower diabetes mortality, whereas dairy and meat did not have a significant association. Three of the articles focused on diabetes mortality were rated as high quality, and 1 article was rated as moderate (Supplemental Table 2). Additional meta-analyses are presented in Supplemental Tables 4–6 and Supplemental Figure 29.

### Metabolic syndrome

The studies on metabolic syndrome incidence were conducted from 2014 to 2021 (Supplemental Table 8). For the most comprehensive and recent meta-analyses, the median number of participants was 19,897 with 6870 cases; however, the number of events for some food groups was not reported. We did not find a meta-analysis on the per-serving comparison of the intake of eggs, legumes, and processed meat and the incidence of metabolic syndrome. The reported heterogeneity for most studies was >0.5, indicating a high diversity in the results. However, for fruits (per serving), nuts (per serving), and white meat (high vs. low), the reported heterogeneity was null, whereas for vegetables (high vs. low), it was 0.30. All the meta-analyses included cohort studies, whereas 3 of them also included case–control studies [44,82]. The median number of included studies for each meta-analysis was 4, ranging from 2 to 9.

The meta-analysis on high-versus-low (Supplemental Figure 30) and per-serving comparisons (Supplemental Figure 31) indicated that a high intake of fish, dairy, nuts, and white meat is associated with a lower risk of metabolic syndrome. In contrast, a high intake of processed meat and red meat is associated with a higher risk of metabolic syndrome. Although the high-versus-low comparison for vegetables showed a reduction in risk of metabolic syndrome, the per-serving comparison was in the same direction and nonsignificant. The high-versus-low comparison of intake of fruits showed a reduction in risk of metabolic syndrome, whereas the per-serving comparison showed no clear associations. The high-versus-low comparison of the intake of sugar-sweetened beverages showed an increase in risk of metabolic syndrome, whereas the per-serving comparison was not significant. Furthermore, a higher intake of whole grains and legumes tended toward a lower risk of metabolic syndrome, whereas the intake of eggs had no clear association.

Among 17 articles focusing on metabolic syndrome incidence, 6 were rated as high quality using AMSTAR-2 scoring; 3 were rated as moderate, 1 as low, and 7 as critically low (Supplemental Table 2). Most frequently, the scores were downgraded due to not reporting the funding sources of the included cohorts, not registering a protocol for the review or using a reporting framework, not using tools for assessing bias and confounding, and not having duplicate study screening. The most comprehensive and recent studies were mostly rated as high/moderate quality, whereas the studies on intake of dairy products (both comparisons), fish (high vs. low), nuts (high vs. low), processed meat, red meat, and white meat were rated as low quality. Additional meta-analyses are presented in Supplemental Tables 4–6 and Supplemental Figures 32–34.

### Certainty of the evidence

The quality of metaevidence for risk of type 2 diabetes was rated moderate for most of the food groups (total dairy, fish, fruits, vegetables, red meat, refined grains, and sugar-sweetened beverages); low to moderate for eggs, white meat, processed meat, legumes, and whole grains; and low for nuts (Table 1 and Supplemental Table 3).

For risk of diabetes-related mortality, the quality of metaevidence was low for most of the food groups (total dairy, fruits, vegetables, meat, nuts, and per-serving comparison for fish), whereas it was moderate for whole grains and high-versus-low comparison for fish (Supplemental Tables 3 and 7).

Similarly, the quality of metaevidence for risk of metabolic syndrome was rated low for most of the food groups (fish, fruits, legumes, nuts, processed meat, red meat, refined grains, whole grains, white meat, and per-serving comparison for total dairy, vegetables, and sugar-sweetened beverages), whereas it was rated very low for eggs. There were 3 food groups with moderate ratings for quality of metaevidence: high-versus-low comparison of dairy and per-serving comparison of vegetables and sugar-sweetened beverages (Supplemental Tables 3 and 8).

### Discussion

In this umbrella review, we systematically examined the associations between food groups and the incidence of type 2 diabetes, the risk of mortality from diabetes, and the incidence of metabolic syndrome. Our findings support the benefits linked to higher consumption of whole grains, which is associated with a reduced risk of type 2 diabetes. Similar nonsignificant trends were also seen for fruits and vegetables. In contrast, a higher intake of red meat, processed meat, and sugar-sweetened beverages is associated with a higher risk of type 2 diabetes. The same associations were seen in the risk of metabolic syndrome and the risk of mortality related to type 2 diabetes.

The only estimate with high confidence according to the NutriGrade score was the association between processed meat and risk of type 2 diabetes, meaning it is likely that the estimate will not change with further research. Meanwhile, the moderate confidence found for most food groups implies that further research might change the estimate. In contrast, further research will provide important evidence in cases where the NutriGrade score was low, and the estimate will likely change. This was the case for most of the food groups and the risk of diabetes-related mortality and metabolic syndrome. The low confidence was mostly due to the low number of available cohort studies, which would reduce confidence when it comes to investigating heterogeneity, publication bias, and precision.

The protective effect of whole grains on the risk of type 2 diabetes is in line with previous studies indicating that long-term intake of whole grains lowers the fasting glucose concentration compared with similar refined food items in a healthy population [107]. The links between whole grains and glycemic control could be explained by the high content of soluble dietary fibers that contribute to increasing the viscosity and thereby slowing the gastric emptying of chyme to the intestine, blunting the glycemic response of meals [108]. Similar to whole grains, fruits and vegetables have complex carbohydrate structures with a high content of dietary fibers [109], but only a borderline reduced risk was found for their intake and the incidence of diabetes. This could be related to the fact that glycemic load differs much between various types of fruits and vegetables and preparation methods (raw/cooked/mashed), which could explain our findings [110]. For example, berries, green leafy vegetables, and cruciferous vegetables are abundant in antioxidants, which can improve insulin sensitivity and lower the risk of metabolic syndrome and type 2 diabetes [75,100]. Moreover, the inclusion of fruit juice in the fruits category in the included cohort studies can be one reason for heterogeneity across the results. The same pattern was seen for nuts and legumes, showing a borderline lower risk with high heterogeneity. However, a network meta-analysis of clinical trials has reported that a higher intake of nuts, legumes, and whole grains was linked to improved lipid profile, suggesting a protective role in the prevention of cardiometabolic disorders [111,112].

In contrast, the intake of sugar-sweetened beverages was strongly associated with an increased risk of metabolic syndrome and type 2 diabetes. Sugar-sweetened beverages usually contain high-fructose syrup, sucrose, or similar simple sugars. These simple sugars are linked with an increased glycemic index, which again is linked to metabolic syndrome and type 2 diabetes [113]. Additionally, studies have shown that the consumption of these types of sugars in liquid form is linked to appetite deregulation, contributing to unhealthy eating behaviors such as emotional eating [[114], [115], [116]]. Specifically, it has been suggested that fructose increases visceral adipose deposition and lowers insulin sensitivity [117]. However, recent systematic reviews investigating the effect of different sweeteners on glycemic response did not find conclusive evidence indicating fructose is more harmful compared with similar amounts of other sugars like sucrose or glucose [118,119].

The consumption of unprocessed and processed meat is linked to obesity, which is often considered a mediator in the causal pathway of type 2 diabetes [120,121]. The nonlinear curves indicate an approximately doubled risk of type 2 diabetes when increasing the intake of processed meat from 0 to 150 g/d [32]. This aligns with studies showing that excessive animal protein consumption is associated with metabolic syndrome and type 2 diabetes [32,37,72,96]. However, this seems to be less of the case for the consumption of white meat. This could be related to both differences in energy density and fatty acid composition as well as differences in preparations, as frying may lead to consuming more fatty acids alongside the meal, whereas steaming and boiling may result in a healthier meal [32].

The consumption of total dairy products did not have a clear association with risk of type 2 diabetes; however, the nonlinear dose–response curve indicated a decreased risk of an intake of up to half a liter of milk per day [65]. Moreover, low-fat dairy products had a borderline inverse association, whereas high-fat dairy products had no association [65]. In the subgroup analysis of dairy products, a reverse association was seen with yogurt, which is in line with previous studies showing the health effects of fermented dairy products on glucose levels [32]. Similarly, the intake of eggs had no clear association with risk of type 2 diabetes. However, the dose–response curve showed an increased risk when increasing the intake to 1 egg or more per day [65]. The studies conducted in the United States generally reported stronger associations between the intake of eggs and risk of type 2 diabetes, which could be related to the meals that usually contain eggs in that region. For example, fried eggs with bacon are a common meal in the United States, whereas in Asia, eggs are often consumed boiled or with vegetables [32,65]. A similar pattern was seen for fish products, which also could be related to frequent meals with fish in the United States, such as fish and chips (fried fish with added oils), whereas in Asia, fish products are frequently consumed baked or with soup [32,65].

The associations between food groups and diabetes-related mortality were generally similar to the associations for the incidence of type 2 diabetes and metabolic syndrome. However, the inverse associations between diabetes-related mortality and nuts and fish were not seen for diabetes incidence. This might be explained by these food groups being energy dense but with a beneficial cardiovascular disease profile [112,122].

In the current umbrella review, we have presented the associations of each included food group with risk of type 2 diabetes; however, combining these food groups into dietary patterns can provide an opportunity to compare individual consumption patterns to dietary recommendations. A large cohort study has suggested a protective role of a diet with higher plant-based intake compared with animal-based intake for incidence of type 2 diabetes [123]. Similarly, another study reported that converting to a vegetarian diet from a nonvegetarian diet was associated with a lower risk of diabetes [124]. Moreover, adherence to Mediterranean diet (which includes high daily consumption of vegetables and whole grain breads, fruits as dessert, olive oil, and nuts and seeds as the primary source of fat, low to moderate consumption of dairy products, mainly cheese and yogurt, moderate intake of fish, white meat and egg, and low consumption of red meat) is inversely associated with incidence of type 2 diabetes [125]. The Mediterranean diet also includes a moderate intake of wine, commonly with meals. A recent umbrella review has reported that low and moderate alcohol consumption is associated with a lower risk of incidence of type 2 diabetes [126,127].

This study has several strengths and limitations. This is the most comprehensive umbrella review to date on the relationship between various food groups and the incidence of type 2 diabetes, incidence of metabolic syndrome, and risk of mortality in people with type 2 diabetes. No other umbrella reviews are currently available with the same scope. We adhered to a preregistered PROSPERO protocol and complied with PRISMA criteria, assuring methodological rigor. However, some information might have been overlooked due to indexing restrictions in databases such as MEDLINE, Embase, Web of Science, and Epistemonikos, or imprecise indicators of relevance in specific article titles and abstracts. The significant heterogeneity across studies indicates that not all food products falling into the same category have the same association with risk of type 2 diabetes and metabolic syndrome. This calls for a need for more detailed food categorization when looking at the same outcome. The most frequent flaw in the quality of the included meta-analyses was the failure to disclose funding for included studies. Furthermore, the absence of a registered protocol or reporting framework could lead to arbitrary decisions, such as when it came to thresholds for study inclusion and exclusion. Moreover, the NutriGrade scoring system used to assess the certainty of the metaevidence is validated and has been used in similar research, but the use of other tools could have provided different results. Additionally, although we exclusively selected publications published in English, we did not find relevant articles in our search records in other languages throughout the screening of abstracts and titles.

In conclusion, a high consumption of whole grains is associated with a reduced risk of type 2 diabetes and metabolic syndrome, with some similar tendencies also for fruits and vegetables. In contrast, high intakes of sugar-sweetened beverages and red and processed meats are associated with an increased risk of type 2 diabetes and metabolic syndrome. The heterogeneity between the studies included in the reviewed meta-analysis, both for high-versus-low and per-serving comparisons, was high, indicating a need for further investigations into subcategories of food groups and risk of type 2 diabetes.

## Author contributions

The authors’ responsibilities were as follows – RLB, EJA, ATO: led screening and data extractions; EJA, MP, RB: conducted the analysis, EJA, RLB: drafted the first draft of the manuscripts; LTF: supervised the study; and all authors contributed to conceiving and designing the study, read and approved the final manuscript, and had full access to the data and take responsibility for the integrity of the data and the accuracy of the data analysis.

## Data availability

Data are available in supplementary data file.

## Funding

The authors were funded by their respective institutions. EJA, ATO, and LMT were funded by Western Norway Regional Health Authority (“Strategiske forskningsmidler”) through the ATLAS4LAR-project. RB was funded by the Foundation Dam (grant number SDAM_FOR462258) in collaboration with the patient organization LHL and co-funding from the University of Bergen. MP and AL were funded with the co-financing of the Italian Ministry of University and Research in the framework of PNC “DARE – Digital lifelong prevention project” (PNC0000002 – CUP B53C22006450001). The views and opinions expressed are solely those of the authors and do not necessarily reflect those of the European Union, nor can the European Union be held responsible for them. The funders had no role in study design, data collection and analysis, decision to publish, or preparation of the manuscript.

## Conflict of interest

All authors have no competing interests.
