# Supplementary material for: Umbrella Review of Systematic Reviews and Meta-Analyses on Consumption of Different Food Groups and Risk of Type 2 Diabetes Mellitus and Metabolic Syndrome
Source: J Nutr. 2025 Mar 22;155(5):1285–97. doi: 10.1016/j.tjnut.2025.03.021 (PMC12121416; doi:10.1016/j.tjnut.2025.03.021)

**Supplemental Material**

Contents

[Supplementary Text 1: Search string 15.05.2024 4](#_Toc192510362)

[Supplementary Table 1: Table of extracted data from all included studies for associations between food groups and incidence of diabetes, diabetes mortality, and incidence of metabolic syndrome as well as a list of excluded studies. 7](#_Toc192510363)

[Supplementary Table 2: Table of quality scores for using AMSTAR-2 quality assessment with 16 items and overall assessment for all included studies. 7](#_Toc192510364)

[Supplementary Table 3: Details of NutriGrade scoring for the most comprehensive/up-to-date meta-analyses on associations between food groups and incidence of type 2 diabetes, diabetes-related mortality and incidence of metabolic syndrome. 8](#_Toc192510365)

[Supplementary Table 4: Table of extracted data for high-versus-low comparisons categorized per food group and ranked by comprehensiveness for associations between food groups and incidence of diabetes type 2, diabetes mortality, and incidence of metabolic syndrome. 10](#_Toc192510366)

[Supplementary Table 5: Table of extracted data for per-serving comparisons categorized per food group and ranked by comprehensiveness for associations between food groups and incidence of diabetes type 2, diabetes mortality, and incidence of metabolic syndrome. 13](#_Toc192510367)

[Supplementary Table 6: Table of extracted data for dose-response comparisons categorized per food for associations between food groups and incidence of diabetes, diabetes mortality, and incidence of metabolic syndrome. 16](#_Toc192510368)

[Supplementary Table 7: Overview of the most comprehensive/up-to-date meta-analyses on associations between food groups and diabetes-related mortality with details on first author, search year, study quality, exposure, number of studies, participants, events, result and certainty of evidence. 18](#_Toc192510369)

[Supplementary Table 8: Overview of the most comprehensive/up-to-date meta-analyses on associations between food groups and incidence of metabolic syndrome with details on first author, search year, study quality, exposure, number of studies, participants, events, results and certainty of evidence. 19](#_Toc192510370)

[Supplementary Figures 1- 26: Figures presenting estimates for studies for each of the food groups and incidence of diabetes type 2 20](#_Toc192510371)

[Supplementary Figures S1: Associations between intake of whole grains for high versus low and incidence of diabetes type 2 from all meta-analyses 20](#_Toc192510372)

[Supplementary Figures S2: Associations between intake of whole grains per serving and incidence of diabetes type 2 from all meta-analyses 21](#_Toc192510373)

[Supplementary Figures S3: Associations between intake of refined grains for high versus low and incidence of diabetes type 2 from all meta-analyses 22](#_Toc192510374)

[Supplementary Figures S4: Associations between intake of refined grains per serving and incidence of diabetes type 2 from all meta-analyses 23](#_Toc192510375)

[Supplementary Figures S5: Associations between intake of fruits for high versus low and incidence of diabetes type 2 from all meta-analyses 24](#_Toc192510376)

[Supplementary Figures S6: Associations between intake of fruits per serving and incidence of diabetes type 2 from all meta-analyses 25](#_Toc192510377)

[Supplementary Figures S7: Associations between intake of vegetables for high versus low and incidence of diabetes type 2 from all meta-analyses 26](#_Toc192510378)

[Supplementary Figures S8: Associations between intake of vegetables per serving and incidence of diabetes type 2 from all meta-analyses 27](#_Toc192510379)

[Supplementary Figures S9: Associations between intake of nuts for high versus low and incidence of diabetes type 2 from all meta-analyses 28](#_Toc192510380)

[Supplementary Figures S10: Associations between intake of nuts per serving and incidence of diabetes type 2 from all meta-analyses 29](#_Toc192510381)

[Supplementary Figures S11: Associations between intake of legumes for high versus low and incidence of diabetes type 2 from all meta-analyses 30](#_Toc192510382)

[Supplementary Figures S12: Associations between intake of legumes per serving and incidence of diabetes type 2 from all meta-analyses 31](#_Toc192510383)

[Supplementary Figures S13: Associations between intake of fish and fish products for high versus low and incidence of diabetes type 2 from all meta-analyses 32](#_Toc192510384)

[Supplementary Figures S14: Associations between intake of fish and fish products per serving and incidence of diabetes type 2 from all meta-analyses 33](#_Toc192510385)

[Supplementary Figures S15: Associations between intake of eggs for high versus low and incidence of diabetes type 2 from all meta-analyses 34](#_Toc192510386)

[Supplementary Figures S16: Associations between intake of eggs per serving and incidence of diabetes type 2 from all meta-analyses 35](#_Toc192510387)

[Supplementary Figures S17: Associations between intake of dairy products for high versus low and incidence of diabetes type 2 from all meta-analyses 36](#_Toc192510388)

[Supplementary Figures S18: Associations between intake of dairy products per serving and incidence of diabetes type 2 from all meta-analyses 37](#_Toc192510389)

[Supplementary Figures S19: Associations between intake of processed meat for high versus low and incidence of diabetes type 2 from all meta-analyses 38](#_Toc192510390)

[Supplementary Figures S20: Associations between intake of processed meat per serving and incidence of diabetes type 2 from all meta-analyses 39](#_Toc192510391)

[Supplementary Figures S22: Associations between intake of red meat per serving and incidence of diabetes type 2 from all meta-analyses 41](#_Toc192510392)

[Supplementary Figures S23: Associations between intake of white meat for high versus low and incidence of diabetes type 2 from all meta-analyses 42](#_Toc192510393)

[Supplementary Figures S24: Associations between intake of white meat per serving and incidence of diabetes type 2 from all meta-analyses 43](#_Toc192510394)

[Supplementary Figures S25: Associations between intake of sugar-sweetened beverages for high versus low and incidence of diabetes type 2 from all meta-analyses 44](#_Toc192510395)

[Supplementary Figures S26: Associations between intake of sugar-sweetened beverages per serving and incidence of diabetes type 2 from all meta-analyses 45](#_Toc192510396)

[Supplementary Figure 27: Associations between food groups (high vs. low consumption) and diabetes type 2 mortality in most comprehensive and up-to-date meta-analyses 46](#_Toc192510397)

[Supplementary Figure 28: Associations between food groups (per serving) and diabetes type 2 mortality in most comprehensive and up-to-date meta-analyses 47](#_Toc192510398)

[Supplementary Figures S29: Associations between intake of whole grains per serving and mortality of diabetes type 2 from all meta-analyses 48](#_Toc192510399)

[Supplementary Figure 30: Associations between food groups (high vs. low consumption) and metabolic syndrome incidence in most comprehensive and up-to-date meta-analyses 49](#_Toc192510400)

[Supplementary Figure 31: Associations between food groups (per serving) and metabolic syndrome incidence in most comprehensive and up-to-date meta-analyses 50](#_Toc192510401)

[Supplementary Figures S32: Associations between intake of dairy products for high versus low and incidence of metabolic syndrome from all meta-analyses 51](#_Toc192510402)

[Supplementary Figures S33: Associations between intake of dairy products per serving and incidence of metabolic syndrome from all meta-analyses 52](#_Toc192510403)

[Supplementary Figures S34: Associations between intake of sugar-sweetened beverages for high versus low and incidence of metabolic syndrome from all meta-analyses 53](#_Toc192510404)

# Supplementary Text 1: Search string 15.05.2024

| Ovid MEDLINE  1 food/ or bread/ or exp dairy products/ or exp dietary carbohydrates/ or exp dietary fats/ or exp dietary proteins/ or exp eggs/ or flour/ or food, processed/ or fruit/ or exp meat/ or molasses/ or nuts/ or seeds/ or exp edible grain/ or exp vegetables/ 628516  2 (food group* or bread* or grain* or cereal* or dairy product* or milk or cheese or yogurt or yoghurt or dietary carbohydrate* or dietary fat* or oil or oils or dietary protein* or egg or eggs or flour or processed food* or fruit or fruits or berry or berries or citrus or meat* or fish or sea food* or chicken or molasses or sugar or sugars or sugar-sweetened beverage* or nut or nuts or seed or seeds or vegetable* or legume* or peas or soy or pulses or bean* or lentil*).ti,ab,kf. 1491357  3 1 or 2 1779884  4 exp Diabetes Mellitus/ or Metabolic Syndrome/ 558210  5 (diabetes or diabetic* or dmt2 or t2dm or dmtII or tIIdm or NIDDM or metabolic syndrome or dysmetabolic syndrome or cardiometabolic syndrome or metabolic cardiovascular syndrome or metabolic x syndrome or syndrome X or Reaven syndrome or insulin resistan*).ti,ab,kf. 906392  6 4 or 5 968602  7 eating/ or drinking/ or food preferences/ 86061  8 (intake* or consum* or eat* or diet*).ti,ab,kf. 1476897  9 7 or 8 1502705  10 3 and 6 and 9 31654  11 meta-analysis/ or "systematic review"/ 347471  12 ((systematic* adj3 (review* or overview*)) or (integrative adj3 (review* or overview*)) or (collaborative adj3 (review* or overview*)) or (meta analy* or metanaly* or meta-analy* or metaanaly* or systematic review*)).ti,ab,kf. 501961  13 11 or 12 531153  14 10 and 13 1322 |
| --- |
| Embase 1 food/ or exp bakery product/ or exp bran/ or exp dairy product/ or exp edible oil/ or exp egg/ or fat/ or exp flour/ or exp food grain/ or exp fruit/ or margarine/ or exp meat/ or exp noodle/ or exp nut/ or pasta/ or exp poultry product/ or exp processed food/ or exp sea food/ or exp soy food/ or exp vegetable/ or exp whole food/ 891276  2 (food group* or bread* or grain* or cereal* or dairy product* or milk or cheese or yogurt or yoghurt or dietary carbohydrate* or dietary fat* or oil or oils or dietary protein* or egg or eggs or flour or processed food* or fruit or fruits or berry or berries or citrus or meat* or fish or sea food* or chicken or molasses or sugar or sugars or sugar-sweetened beverage* or nut or nuts or seed or seeds or vegetable* or legume* or peas or soy or pulses or bean* or lentil*).ti,ab,kf. 1647015  3 1 or 2 2048476  4 food intake/ or drinking/ or eating/ or fish consumption/ or fruit consumption/ or meat consumption/ or vegetable consumption/ 230182  5 (intake* or consum* or eat* or diet*).ti,ab,kf. 1869456  6 4 or 5 1924684  7 exp diabetes mellitus/ or metabolic syndrome X/ 1327577  8 (diabetes or diabetic* or dmt2 or t2dm or dmtII or tIIdm or NIDDM or metabolic syndrome or dysmetabolic syndrome or cardiometabolic syndrome or metabolic cardiovascular syndrome or metabolic x syndrome or syndrome X or Reaven syndrome or insulin resistan*).ti,ab,kf. 1369891  9 7 or 8 1641124  10 3 and 6 and 9 44491  11 exp meta-analysis/ or "systematic review"/ 601300  12 ((systematic* adj3 (review* or overview*)) or (integrative adj3 (review* or overview*)) or (collaborative adj3 (review* or overview*)) or (meta analy* or metanaly* or meta-analy* or metaanaly* or systematic review*)).ti,ab,kf. 626231  13 11 or 12 769560  14 10 and 13 2124 |
| Epistemonikos (Epistemonikos Foundation) Title/abstract-searches:  ("food group" or "foods groups" or bread* or grain* or cereal* or "dairy product" or "dairy products" or milk or cheese or yogurt or yoghurt or "dietary carbohydrate" or "dietary carbohydrates" or "dietary fat" or "dietary fats" or oil or oils or "dietary protein" or "dietary proteins" or egg or eggs or flour or "processed food" or "processed foods" or fruit or fruits or berry or berries or citrus or meat* or fish or "sea food" or "sea foods" or chicken or molasses or sugar or sugars or "sugar-sweetened beverage" or "sugar-sweetened beverages" or nut or nuts or seed or seeds or vegetable* or legume* or peas or soy or pulses or bean* or lentil*)  AND  (intake* or consum* or eat* or diet*)  AND  (diabetes or diabetic* or dmt2 or t2dm or dmtII or tIIdm or NIDDM or "metabolic syndrome" or "dysmetabolic syndrome" or "cardiometabolic syndrome" or "metabolic cardiovascular syndrome" or "metabolic x syndrome" or "syndrome X" or "Reaven syndrome" or "insulin resistance" or "insulin resistant")  Broad synthesis: 72  Systematic reviews: 797  Total: 869 |
| Web of Science Core Collection # Entitlements:  - WOS.SCI: 1945 to 2024  - WOS.AHCI: 1975 to 2024  - WOS.ESCI: 2019 to 2024  - WOS.SSCI: 1956 to 2024  # Searches:  #1 Search: TS=((("food group*" or bread* or grain* or cereal* or "dairy product*" or milk or cheese or yogurt or yoghurt or "dietary carbohydrate*" or "dietary fat*" or oil or oils) NEAR/2 (intake* or consum* or eat* or diet*))) Results: 80906  #2 Search: TS=((("dietary protein*" or egg or eggs or flour or "processed food*" or fruit or fruits or berry or berries or citrus or meat* or fish or "sea food*" or chicken or molasses) NEAR/2 (intake* or consum* or eat* or diet*))) Results: 109370  #3 Search: TS=(((sugar or sugars or "sugar-sweetened beverage*" or nut or nuts or seed or seeds or vegetable* or legume* or peas or soy or pulses or bean* or lentil*) NEAR/2 (intake* or consum* or eat* or diet*))) Results: 54739  #4 Search: #1 OR #2 OR #3 Results: 213484  #5 Search: TS=((diabetes or diabetic* or dmt2 or t2dm or dmtII or tIIdm or NIDDM or "metabolic syndrome" or "dysmetabolic syndrome" or "cardiometabolic syndrome" or "metabolic cardiovascular syndrome" or "metabolic x syndrome" or "syndrome X" or "Reaven syndrome" or "insulin resistan*")) Results: 1111917  #6 Search: TI=(((systematic* NEAR/2 (review* or overview*)) or (integrative NEAR/2 (review* or overview*)) or (collaborative NEAR/2 (review* or overview*)) or ("meta analy*" or metanaly* or meta-analy* or metaanaly* or "systematic review*"))) Results: 436388  #7 Search: #4 AND #5 AND #6 Results: 759 |

# **Supplementary Table 1:** Table of extracted data from all included studies for associations between food groups and incidence of diabetes, diabetes mortality, and incidence of metabolic syndrome as well as a list of excluded studies.

-Provided in a separate file as a spreadsheet

# Supplementary Table 2: **Table of quality scores for using AMSTAR-2 quality assessment with 16 items and overall assessment for all included studies.**

-Provided in a separate file as a spreadsheet

# Supplementary Table 3: Details of NutriGrade scoring for the most comprehensive/up-to-date meta-analyses on associations between food groups and incidence of type 2 diabetes, diabetes-related mortality and incidence of metabolic syndrome.

| Autor  (Year) | Food group | Comparison type | Risk of bias (2P) | Precision (1P) | Directness (1P) | Heterogeneity (1P) | Publication bias (1P) | Funding bias (1P) | Effect size (1P) | Dose response (1P) | Sum | NutriGrade |
| --- | --- | --- | --- | --- | --- | --- | --- | --- | --- | --- | --- | --- |
| Schwingshackl (2017) | Dairy | HL | 1.25 | 1 | 1 | 0.8 | 1 | 1 | 0 | 1 | 7.05 | Moderate |
| Schwingshackl (2017) | Dairy | PS (200g) | 1.25 | 1 | 1 | 0.8 | 0.5 | 1 | 0 | 1 | 6.55 | Moderate |
| Schwingshackl (2017) | Eggs | HL | 1.25 | 0 | 1 | 0.8 | 1 | 1 | 0 | 0 | 5.05 | Low |
| Fan (2019) | Eggs | PS (50g) | 2 | 1 | 1 | 0.6 | 1 | 0.5 | 0 | 1 | 7.1 | Moderate |
| Fan (2019) | Fish | HL | 2 | 1 | 1 | 0.8 | 0.5 | 0.5 | 0 | 1 | 6.8 | Moderate |
| Fan (2019) | Fish | PS (100g) | 2 | 1 | 1 | 0.3 | 0.5 | 0.5 | 0 | 1 | 6.3 | Moderate |
| Halvorsen (2020) | Fruit | HL | 1 | 1 | 1 | 0.8 | 1 | 1 | 0 | 1 | 6.8 | Moderate |
| Halvorsen (2020) | Fruit | PS (80g) | 1 | 1 | 1 | 0.8 | 1 | 1 | 0 | 1 | 6.8 | Moderate |
| Thorisdottir (2022) | Legumes | HL | 1 | 1 | 1 | 0.6 | 1 | 0.5 | 0 | 0 | 5.1 | Low |
| Schwingshackl (2017) | Legumes | PS (50g) | 1.25 | 1 | 1 | 0.8 | 1 | 1 | 0 | 0 | 6.05 | Moderate |
| Schwingshackl (2017) | Nuts | HL | 1.25 | 1 | 1 | 0.4 | 0.5 | 1 | 0 | 0 | 5.15 | Low |
| Schwingshackl (2017) | Nuts | PS (28g) | 1.25 | 1 | 1 | 0.4 | 0.5 | 1 | 0 | 0 | 5.15 | Low |
| Schwingshackl (2017) | Processed meat | HL | 1.25 | 1 | 1 | 0.8 | 1 | 1 | 1 | 1 | 8.05 | High |
| Shi (2022) | Processed meat | PS (50g) | 1.25 | 1 | 1 | 0.6 | 1 | 1 | 1 | 0 | 6.85 | Moderate |
| Zhang (2021) | Red meat | HL | 2 | 1 | 1 | 0.8 | 1 | 1 | 0 | 1 | 7.8 | Moderate |
| Shi (2022) | Red meat | PS (100g) | 1.25 | 1 | 1 | 0.6 | 1 | 1 | 1 | 0 | 6.85 | Moderate |
| Schwingshackl (2017) | Refined grains | HL | 1.25 | 1 | 1 | 0.8 | 1 | 1 | 0 | 0 | 6.05 | Moderate |
| Schwingshackl (2017) | Refined grains | PS (30g) | 1.25 | 1 | 1 | 0.8 | 1 | 1 | 0 | 0 | 6.05 | Moderate |
| Qin (2019) | SSBs | HL | 2 | 1 | 1 | 0.8 | 0.5 | 0.5 | 1 | 1 | 7.8 | Moderate |
| Qin (2019) | SSBs | PS (250g) | 2 | 1 | 1 | 0.8 | 0.5 | 0.5 | 0 | 1 | 6.8 | Moderate |
| Halvorsen (2020) | Vegetables | HL | 1 | 1 | 1 | 0.8 | 1 | 1 | 0 | 1 | 6.8 | Moderate |
| Halvorsen (2020) | Vegetables | PS (100g) | 1 | 1 | 1 | 0.8 | 0.5 | 1 | 0 | 1 | 6.3 | Moderate |
| Ramel (2021) | White meat | HL | 1 | 1 | 1 | 0.2 | 0.5 | 1 | 0 | 0 | 4.7 | Low |
| Yang (2019) | White meat | PS (100g) | 2 | 1 | 1 | 0.8 | 0.5 | 0.5 | 0 | 1 | 6.8 | Moderate |
| Ghanbari-Gohari (2021) | Whole grains | HL | 1 | 1 | 1 | 0.8 | 1 | 1 | 1 | 1 | 7.8 | Moderate |
| Ghanbari-Gohari (2021) | Whole grains | PS (30g) | 1 | 1 | 1 | 0.4 | 0.5 | 1 | 0 | 1 | 5.9 | Low |
| Barbaresko (2022) | Dairy | PS (200g) | 1 | 1 | 1 | 0 | 0 | 1 | 0 | 1 | 4 | Low |
| Barbaresko (2022) | Eggs | PS (50g) | 1 | 1 | 1 | 0.4 | 0.5 | 1 | 1 | 1 | 6.4 | Moderate |
| Jayedi (2019) | Fish | HL | 2 | 1 | 1 | 0.3 | 0.5 | 1 | 0 | 2 | 6.8 | Moderate |
| Barbaresko (2022) | Fish | PS (100g) | 1 | 1 | 1 | 0.4 | 0.5 | 1 | 0 | 1 | 5.9 | Low |
| Barbaresko (2022) | Fruit | PS (80g) | 1 | 1 | 1 | 0 | 0 | 1 | 0 | 1 | 5 | Low |
| Barbaresko (2022) | Meat | PS (50g) | 1 | 1 | 1 | 0 | 0 | 1 | 0 | 1 | 4 | Low |
| Barbaresko (2022) | Nuts | PS (28g) | 1 | 1 | 1 | 0 | 0 | 1 | 0 | 1 | 4 | Low |
| Barbaresko (2022) | Vegetables | PS (100g) | 1 | 1 | 1 | 0 | 0 | 1 | 0 | 1 | 5 | Low |
| Aune (2016) | Whole grains | HL | 2 | 1 | 1 | 0 | 0 | 1 | 1 | 2 | 6.5 | Moderate |
| Barbaresko (2022) | Whole grains | PS (30g) | 1 | 1 | 1 | 0 | 0 | 1 | 1 | 1 | 5.5 | Low |
| Mena-Sanchez (2017) | Dairy | HL | 2 | 1 | 1 | 0.4 | 0.5 | 1 | 1 | 1 | 7.9 | Moderate |
| Kim (2015) | Dairy | PS (200g) | 2 | 1 | 1 | 0 | 0 | 0.5 | 0 | 0 | 4.5 | Low |
| Ding (2021) | Eggs | HL | 1 | 0 | 1 | 0 | 0 | 0.5 | 0 | 0 | 2.5 | Very low |
| Kim (2014) | Fish | HL | 1 | 1 | 1 | 0 | 0 | 1 | 1 | 0 | 5 | Low |
| Karimi (2019) | Fish | PS (100g) | 2 | 1 | 1 | 0 | 0.5 | 1 | 0 | 0 | 5.5 | Low |
| Zhang (2017) | Fruit | HL | 2 | 1 | 1 | 0 | 0 | 1 | 0 | 0 | 5 | Low |
| Lee (2018) | Fruit | PS (80g) | 2 | 1 | 1 | 0 | 0 | 0.5 | 0 | 1 | 5.5 | Low |
| Jiang (2019) | Legumes | HL | 2 | 1 | 1 | 0.4 | 0.5 | 0.5 | 0 | 0 | 5.4 | Low |
| Zhang (2018) | Nuts | HL | 2 | 1 | 1 | 0 | 0.5 | 1 | 0 | 0 | 5.5 | Low |
| Li (2017) | Nuts | PS (28g) | 2 | 1 | 1 | 0 | 0 | 1 | 0 | 0 | 5 | Low |
| Guo (2021) | Processed meat | HL | 1 | 1 | 1 | 0 | 0 | 1 | 1 | 0 | 5 | Low |
| Guo (2021) | Red meat | HL | 1 | 1 | 1 | 0 | 0 | 1 | 1 | 0 | 5 | Low |
| Guo (2021) | Refined grains | HL | 2 | 0 | 1 | 0.3 | 0 | 1 | 0 | 0 | 4.3 | Low |
| Muñoz-Cabrejas (2022) | SSBs | HL | 1.75 | 1 | 1 | 0 | 0.5 | 1 | 0 | 0 | 5.25 | Low |
| Zhang (2014) | SSBs | PS (250g) | 2 | 1 | 1 | 0 | 0.5 | 1 | 0 | 1 | 6.5 | Moderate |
| Zhang (2017) | Vegetables | HL | 2 | 1 | 1 | 0 | 0 | 1 | 0 | 0 | 5 | Low |
| Lee (2018) | Vegetables | PS (100g) | 2 | 1 | 1 | 0 | 0 | 0.5 | 1 | 1 | 6.5 | Moderate |
| Guo (2021) | White meat | HL | 1 | 1 | 1 | 0 | 0 | 1 | 0 | 0 | 4 | Low |
| Guo (2021) | Whole grains | HL | 2 | 1 | 1 | 0 | 0 | 1 | 0 | 0 | 5 | Low |

HL: high versus low. PS: Per serving with serving sizes in the parentheses. SSBs: sugar-sweetened beverages.

# Supplementary Table 4: Table of extracted data for high-versus-low comparisons categorized per food group and ranked by comprehensiveness for associations between food groups and incidence of diabetes type 2, diabetes mortality, and incidence of metabolic syndrome.

| **Food group** | **Author (Year)** | **Studies/Participants/Events** | **AMSTAR-2** | **Results** | **I^2^** | **Most relevant** | **Most recent** | **Most studies** | **Most participants** | **Most cases** | **Best quality** |
| --- | --- | --- | --- | --- | --- | --- | --- | --- | --- | --- | --- |
| Diabetes-related mortality | | | | | | | | | | | |
| Fish | Jayedi (2019) | 8 / 57077 / NA | High | 0.86 [0.76, 0.96] | 0.5 | 1 | 1 | 1 | 1 | NA | 1 |
| Whole grains | Aune (2016) | 4 / 632849 / 808 | Moderate | 0.64 [0.42, 0.98] | 0.64 | 1 | 1 | 1 | 1 | 1 | 1 |
| Incidence of type 2 diabetes | | | | | | | | | | | |
| Dairy | Schwingshackl (2017) | 21 / 566867 / 44474 | Moderate | 0.91 [0.85, 0.97] | 0.63 | 1 | 0 | 1 | 1 | 1 | 1 |
|  | Aune (2013) | 14 / 426055 / 26976 | Low | 0.89 [0.82, 0.96] | 0.42 | 0 | 0 | 0 | 0 | 0 | 0 |
|  | Fan (2019) | 15 / 252552 / 16961 | Low | 0.98 [0.93, 1.02] | 0.27 | 0 | 1 | 0 | 0 | 0 | 0 |
|  | Khoramdad (2016) | 13 / 460421 / 24355 | Low | 0.89 [0.82, 0.97] | 0 | 0 | 0 | 0 | 0 | 0 | 0 |
|  | Tian (2017) | 11 / 282771 / 10381 | Low | 0.89 [0.84, 0.94] | 0.49 | 0 | 0 | 0 | 0 | 0 | 0 |
|  | Tong (2011) | 6 / 263838 / 10808 | Low | 0.86 [0.79, 0.92] | 0.3 | 0 | 0 | 0 | 0 | 0 | 0 |
| Eggs | Schwingshackl (2017) | 13 / 315358 / 17629 | Moderate | 1.08 [0.95, 1.22] | 0.69 | 1 | 0 | 0 | 0 | 0 | 1 |
|  | Djoussé (2015) | 12 / 219979 / 8911 | Low | 1.09 [0.99, 1.2] | NA | 0 | 0 | 0 | 0 | 0 | 0 |
|  | Fan (2019) | 19 / 374891 / 20691 | Low | 1.1 [1.03, 1.16] | 0.7 | 0 | 1 | 1 | 1 | 1 | 0 |
|  | Li (2012) | 7 / 64447 / 4649 | Low | 1.68 [1.41, 2] | 0.25 | 0 | 0 | 0 | 0 | 0 | 0 |
|  | Shin (2012) | 5 / 69297 / 4889 | High | 1.42 [1.09, 1.86] | 0.54 | 0 | 0 | 0 | 0 | 0 | 0 |
|  | Tamez (2015) | 10 / 251213 / 12156 | Low | 1.13 [1.04, 1.22] | NA | 0 | 0 | 0 | 0 | 0 | 0 |
|  | Tian (2017) | 5 / 67796 / 5281 | Low | 1.03 [0.64, 1.67] | 0.91 | 0 | 0 | 0 | 0 | 0 | 0 |
| Fish | Fan (2019) | 20 / 698894 / 43239 | Low | 1.08 [1, 1.18] | 0.77 | 1 | 1 | 1 | 1 | 0 | 1 |
|  | Schwingshackl (2017) | 16 / 637716 / 45029 | Moderate | 1.04 [0.95, 1.13] | 0.76 | 0 | 0 | 0 | 0 | 1 | 0 |
|  | Tian (2017) | 9 / 466166 / 21825 | Low | 1.03 [0.89, 1.17] | 0.8 | 0 | 0 | 0 | 0 | 0 | 0 |
|  | Yang (2019) | 20 / 682622 / 42084 | Low | 1.01 [0.93, 1.1] | 0.75 | 0 | 0 | 1 | 0 | 0 | 0 |
|  | Zhang (2013) | 10 / 549955 / NA | Low | 1.04 [0.89, 1.2] | 0.83 | 0 | 0 | 0 | 0 | NA | 0 |
|  | Zhou (2011) | 9 / 367294 / 18262 | Low | 1.15 [0.98, 1.35] | 0.79 | 0 | 0 | 0 | 0 | 0 | 0 |
| Fruit | Halvorsen (2020) | 20 / 1532167 / 81313 | High | 0.93 [0.9, 0.97] | 0.09 | 1 | 1 | 1 | 1 | 1 | 1 |
|  | Li (2014) | 9 / 424677 / 22995 | High | 0.93 [0.88, 0.99] | 0 | 0 | 0 | 0 | 0 | 0 | 0 |
|  | Li (2013) | 9 / 403259 / 27940 | Low | 0.92 [0.86, 0.97] | 0.38 | 0 | 0 | 0 | 0 | 0 | 0 |
|  | Schwingshackl (2017) | 15 / 1060538 / 70968 | Moderate | 0.96 [0.93, 1] | 0.29 | 0 | 0 | 0 | 0 | 0 | 0 |
|  | Carter (2009) | 5 / 204654 / 8563 | Low | 0.93 [0.83, 1.01] | 0.52 | 0 | 0 | 0 | 0 | 0 | 0 |
|  | Wang (2014) | 13 / 508578 / 33987 | Low | 0.91 [0.87, 0.96] | 0.11 | 0 | 0 | 0 | 0 | 0 | 0 |
| Legumes | Thorisdottir (2022) | 10 / 312874 / 15992 | High | 0.9 [0.77, 1.06] | 0.88 | 1 | 1 | 1 | 0 | 0 | 1 |
|  | Schwingshackl (2017) | 10 / 536860 / 29223 | Moderate | 0.96 [0.87, 1.05] | 0.85 | 0 | 0 | 1 | 1 | 1 | 0 |
|  | Tang (2019) | 7 / 271709 / 11232 | High | 0.95 [0.79, 1.14] | 0.85 | 0 | 0 | 0 | 0 | 0 | 0 |
| Nuts | Schwingshackl (2017) | 8 / 313847 / 27016 | Moderate | 0.95 [0.85, 1.05] | 0.67 | 1 | 0 | 1 | 0 | 1 | 1 |
|  | Arnesen (2021) | 5 / 211091 / 24389 | High | 0.95 [0.75, 1.21] | 0.82 | 0 | 1 | 0 | 0 | 0 | 0 |
|  | Aune (2016) | 4 / 193928 / 800 | High | 0.68 [0.52, 0.9] | 0 | 0 | 0 | 0 | 0 | 0 | 0 |
|  | Becerra-Tomas (2020) | 4 / 194168 / 12878 | Low | 1.04 [0.94, 1.15] | 0.6 | 0 | 0 | 0 | 0 | 0 | 0 |
|  | Guo (2013) | 6 / 263663 / 11580 | Low | 0.98 [0.84, 1.15] | 0.68 | 0 | 0 | 0 | 0 | 0 | 0 |
|  | Luo (2013) | 4 / 2918625 / 12878 | Moderate | 1 [0.84, 1.19] | 0.67 | 0 | 0 | 0 | 1 | 0 | 0 |
|  | Zhou (2013) | 6 / 342213 / 14400 | Low | 0.92 [0.78, 1.09] | 0.79 | 0 | 0 | 0 | 0 | 0 | 0 |
| Processed meat | Schwingshackl (2017) | 14 / 550342 / 43781 | Moderate | 1.27 [1.2, 1.35] | 0.55 | 1 | 0 | 0 | 0 | 0 | 1 |
|  | Aune (2008) | 9 / 380606 / 9999 | Low | 1.41 [1.25, 1.6] | 0.37 | 0 | 0 | 0 | 0 | 0 | 0 |
|  | Fan (2019) | 22 / 749796 / 46939 | Low | 1.25 [1.15, 1.35] | 0.77 | 0 | 0 | 1 | 0 | 0 | 0 |
|  | Micha (2009) | 7 / 1218380 / 10797 | High | 1.19 [1.11, 1.27] | NA | 0 | 0 | 0 | 1 | 0 | 0 |
|  | Tian (2017) | 11 / 408212 / 11990 | Low | 1.39 [1.29, 1.49] | 0.49 | 0 | 0 | 0 | 0 | 0 | 0 |
|  | Yang (2019) | 17 / 663144 / 49086 | Low | 1.25 [1.13, 1.37] | 0.92 | 0 | 0 | 0 | 0 | 1 | 0 |
|  | Zhang (2021) | 7 / 465995 / 31020 | High | 1.27 [1.15, 1.4] | 0.81 | 0 | 1 | 0 | 0 | 0 | 0 |
| Red meat | Zhang (2021) | 14 / 674345 / 50007 | High | 1.15 [1.08, 1.23] | 0.68 | 1 | 1 | 0 | 0 | 1 | 1 |
|  | Aune (2008) | 12 / 433070 / 12226 | Low | 1.21 [1.07, 1.38] | 0.36 | 0 | 0 | 0 | 0 | 0 | 0 |
|  | Fan (2019) | 18 / 657454 / 48400 | Low | 1.22 [1.14, 1.29] | 0.6 | 0 | 0 | 1 | 0 | 0 | 0 |
|  | Micha (2009) | 7 / 1218380 / 10797 | High | 1.16 [0.92, 1.46] | NA | 0 | 0 | 0 | 1 | 0 | 0 |
|  | Schwingshackl (2017) | 15 / 586040 / 45702 | Moderate | 1.21 [1.13, 1.3] | 0.65 | 0 | 0 | 0 | 0 | 0 | 0 |
|  | Tian (2017) | 11 / 408212 / 11990 | Low | 1.22 [1.09, 1.36] | 0.51 | 0 | 0 | 0 | 0 | 0 | 0 |
|  | Yang (2019) | 17 / 663144 / 49086 | Low | 1.22 [1.16, 1.28] | 0.5 | 0 | 0 | 0 | 0 | 0 | 0 |
|  | Zeraatkar (2019) | 12 / 211467 / NA | High | 0.96 [0.9, 1.03] | NA | 0 | 0 | 0 | 0 |  | 0 |
| Refined grains | Schwingshackl (2017) | 14 / 599772 / 22559 | Moderate | 1.01 [0.92, 1.1] | 0.54 | 1 | 1 | 1 | 1 | 1 | 1 |
|  | Aune (2013) | 6 / 258078 / 9545 | Low | 0.94 [0.82, 1.09] | 0.64 | 0 | 0 | 0 | 0 | 0 | 0 |
| SSBs | Qin (2019) | 18 / 1010392 / 34788 | Moderate | 1.27 [1.18, 1.36] | 0.8 | 1 | 0 | 1 | 1 | 1 | 1 |
|  | Li (2022) | 17 / 566178 / 30666 | High | 1.27 [1.17, 1.38] | 0.69 | 0 | 1 | 0 | 0 | 0 | 0 |
|  | Meng (2020) | 17 / 645658 / 30894 | High | 1.29 [1.23, 1.34] | 0.3 | 0 | 0 | 0 | 0 | 0 | 0 |
|  | Santos (2021) | 13 / 706421 / NA | Moderate | 1.2 [1.13, 1.28] | 0.7 | 0 | 0 | 0 | 0 |  | 0 |
|  | Schwingshackl (2017) | 10 / 352937 / 30887 | Moderate | 1.3 [1.2, 1.4] | 0.34 | 0 | 0 | 0 | 0 | 0 | 0 |
|  | Wang (2014) | 8 / 286697 / 29264 | Low | 1.3 [1.21, 1.39] | 0.13 | 0 | 0 | 0 | 0 | 0 | 0 |
| Vegetables | Halvorsen (2020) | 17 / 973814 / 52959 | High | 0.95 [0.88, 1.02] | 0.6 | 1 | 1 | 1 | 1 | 0 | 1 |
|  | Carter (2009) | 5 / 204654 / 8563 | Low | 0.91 [0.76, 1.09] | 0.78 | 0 | 0 | 0 | 0 | 0 | 0 |
|  | Li (2014) | 8 / 290927 / 20933 | High | 0.9 [0.8, 1.01] | 0.66 | 0 | 0 | 0 | 0 | 0 | 0 |
|  | Schwingshackl (2017) | 13 / 944502 / 63299 | Moderate | 0.95 [0.89, 1.01] | 0.59 | 0 | 0 | 0 | 0 | 1 | 0 |
|  | Wang (2014) | 7 / 251235 / 19851 | Low | 0.91 [0.82, 1.01] | 0.57 | 0 | 0 | 0 | 0 | 0 | 0 |
| White meat | Ramel (2021) | 7 / 1241230 / NA | Low | 0.98 [0.87, 1.11] | 0.82 | 1 | 1 | 0 | 1 | NA | 1 |
|  | Fan (2019) | 12 / 432947 / 32812 | Low | 1.04 [1, 1.08] | 0.18 | 0 | 0 | 1 | 0 | 0 | 0 |
|  | Yang (2019) | 11 / 411276 / 32983 | Low | 1 [0.93, 1.07] | 0.68 | 0 | 0 | 0 | 0 | 1 | 0 |
| Whole grains | Ghanbari-Gohari (2021) | 11 / 436282 / 37249 | Moderate | 0.79 [0.73, 0.85] | 0.77 | 1 | 1 | 0 | 0 | 1 | 1 |
|  | Aune (2013) | 10 / 385868 / 19829 | Low | 0.74 [0.71, 0.78] | 0 | 0 | 0 | 0 | 0 | 0 | 0 |
|  | Schwingshackl (2017) | 13 / 514663 / 29633 | Moderate | 0.77 [0.71, 0.84] | 0.86 | 0 | 0 | 1 | 1 | 0 | 0 |
| Incidence of metabolic syndrome | | | | | | | | | | | |
| Dairy | Mena-Sanchez (2017) | 9 / 33001 / 9953 | Low | 0.73 [0.64, 0.83] | 0.62 | 1 | 1 | 1 | 1 | 1 | 1 |
|  | Chen (2014) | 7 / 24217 / NA | Low | 0.86 [0.79, 0.92] | 0 | 0 | 0 | 0 | 0 | NA | 0 |
|  | Kim (2015) | 8 / 31944 / 6870 | Low | 0.85 [0.73, 0.98] | 0.43 | 0 | 0 | 0 | 0 | 0 | 0 |
| Eggs | Ding (2021) | 4 / 15824 / NA | High | 0.99 [0.77, 1.26] | 0.53 | 1 | 1 | 1 | 1 | NA | 1 |
| Fish | Kim (2014) | 2 / 7860 / 1671 | Low | 0.71 [0.58, 0.87] | 0.61 | 1 | 1 | 1 | 1 | 1 | 1 |
| Fruit | Zhang (2017) | 2 / 7379 / NA | High | 0.81 [0.75, 0.88] | 0.62 | 1 | 1 | 1 | 1 | NA | 1 |
| Legumes | Jiang (2019) | 8 / 5643 / 1570 | Moderate | 0.93 [0.77, 1.14] | 0.74 | 1 | 1 | 1 | 1 | 1 | 1 |
| Nuts | Zhang (2018) | 5 / 27304 / NA | Low | 0.84 [0.76, 0.92] | 0.8 | 1 | 1 | 1 | 1 | NA | 1 |
| Processed meat | Guo (2021) | 4 / 5959 / NA | Low | 1.48 [1.11, 1.97] | 0.64 | 1 | 1 | 1 | 1 | NA | 1 |
| Red meat | Guo (2021) | 3 / 5535 / NA | Low | 1.32 [1.14, 1.54] | 0.54 | 1 | 1 | 1 | 1 | NA | 1 |
| Refined grains | Guo (2021) | 7 / 23985 / NA | High | 1.1 [0.86, 1.4] | 0.75 | 1 | 1 | 1 | 1 | NA | 1 |
| SSBs | Muñoz-Cabrejas (2022) | 5 / 28932 / 7667 | Moderate | 1.18 [1.06, 1.32] | 0.7 | 1 | 1 | 0 | 1 | 0 | 1 |
|  | Narain (2015) | 2 / 15553 / NA | Low | 1.22 [0.86, 1.72] | 0.53 | 0 | 0 | 0 | 0 | NA | 0 |
|  | Zhang (2014) | 7 / 28778 / 8033 | Moderate | 1.16 [1.07, 1.25] | 0.34 | 0 | 0 | 1 | 0 | 1 | 0 |
| Vegetables | Zhang (2017) | 3 / 8267 / NA | High | 0.89 [0.85, 0.93] | 0.3 | 1 | 1 | 1 | 1 | NA | 1 |
| White meat | Guo (2021) | 3 / 7270 / NA | Low | 0.85 [0.75, 0.97] | 0 | 1 | 1 | 1 | 1 | NA | 1 |
| Whole grains | Guo (2021) | 4 / 24115 / NA | High | 0.91 [0.74, 1.12] | 0.65 | 1 | 1 | 1 | 1 | NA | 1 |

SSBs: sugar-sweetened beverages.

# Supplementary Table 5: Table of extracted data for per-serving comparisons categorized per food group and ranked by comprehensiveness for associations between food groups and incidence of diabetes type 2, diabetes mortality, and incidence of metabolic syndrome.

| **Food group** | **Author (Year)** | **Studies/Participants/Events** | **AMSTAR-2** | | **Results*** | **I^2^** | **Most relevant** | **Most recent** | **Most studies** | **Most participants** | **Most cases** | **Best quality** |
| --- | --- | --- | --- | --- | --- | --- | --- | --- | --- | --- | --- | --- |
| Diabetes-related mortality | | | | | | | | | | | | |
| Dairy | Barbaresko (2022) | 4 / 14493 / 1913 | High | 1.00 [0.96, 1.08] | | 0 | 1 | 1 | 1 | 1 | 1 | 1 |
| Eggs | Barbaresko (2022) | 7 / 64545 / 3087 | High | 1.28 [1.10, 1.40] | | 0.56 | 1 | 1 | 1 | 1 | 1 | 1 |
| Fish | Barbaresko (2022) | 6 / 24854 / 3835 | High | 0.95 [0.92, 0.99] * | | 0 | 1 | 1 | 1 | 1 | 1 | 1 |
| Fruit | Barbaresko (2022) | 4 / 65164 / 6613 | High | 0.85 [0.78, 0.94] | | 0.68 | 1 | 1 | 1 | 1 | 1 | 1 |
| Meat | Barbaresko (2022) | 3 / 13610 / 1426 | High | 1.03 [0.91, 1.15] * | | 0 | 1 | 1 | 1 | 1 | 1 | 1 |
| Nuts | Barbaresko (2022) | 2 / 22601 / 6512 | High | 0.96 [0.92, 1.01] * | | 0.84 | 1 | 1 | 1 | 1 | 1 | 1 |
| Vegetables | Barbaresko (2022) | 2 / 16662 / 1862 | High | 0.88 [0.82, 0.94] * | | 0 | 1 | 1 | 1 | 1 | 1 | 1 |
| Whole grains | Barbaresko (2022) | 2 / 14035 / 1368 | High | 0.77 [0.60, 0.99] | | 0 | 1 | 1 | 0 | 0 | 1 | 1 |
|  | Li (2015) | 2 / 487451 / 465 | High | 0.91 [0.76, 1.11] * | | 0.84 | 0 | 0 | 0 | 0 | 0 | 1 |
|  | Aune (2016) | 4 / 632849 / 808 | Moderate | 0.79 [0.61, 1.02] | | 0.85 | 0 | 0 | 1 | 1 | 0 | 0 |
| Incidence of type 2 diabetes | | | | | | | | | | | | |
| Dairy | Schwingshackl (2017) | 21 / 566872 / 44474 | Moderate | 0.97 [0.94, 0.99] * | | 0.74 | 1 | 0 | 0 | 0 | 1 | 0 |
|  | Aune (2013) | 12 / 426055 / 26976 | Low | 0.96 [0.93, 0.99] | | 0.33 | 0 | 0 | 0 | 0 | 0 | 0 |
|  | Fan (2019) | 7 / 98606 / 5662 | Low | 1.02 [1.00, 1.06] | | 0.05 | 0 | 0 | 0 | 0 | 0 | 0 |
|  | Feng (2021) | 20 / 560852 / 36291 | High | 0.97 [0.95, 0.99] * | | 0.58 | 0 | 1 | 0 | 0 | 0 | 1 |
|  | Gijsbers (2015) | 16 / 489113 / 38993 | Low | 0.97 [0.95, 1.00] * | | 0.66 | 0 | 0 | 0 | 0 | 0 | 0 |
|  | Soedamah-Muthu (2018) | 22 / 579832 / 43118 | Low | 0.97 [0.95, 1.00] * | | 0.63 | 0 | 0 | 1 | 1 | 0 | 0 |
|  | Tong (2011) | 3 / 119623 / 4810 | Low | 0.94 [0.92, 0.97] * | | NA | 0 | 0 | 0 | 0 | 0 | 0 |
| Eggs | Fan (2019) | 19 / 374891 / 20691 | Low | 1.01 [0.99, 1.03] * | | 0.54 | 1 | 1 | 1 | 1 | 1 | 0 |
|  | Schwingshackl (2017) | 13 / 315358 / 17629 | Moderate | 1.14 [0.92, 1.39] | | 0.77 | 0 | 0 | 0 | 0 | 0 | 1 |
| Fish | Fan (2019) | 8 / 698894 / 43239 | Low | 0.98 [0.85, 1.14] | | 0.61 | 1 | 1 | 0 | 1 | 1 | 0 |
|  | Schwingshackl (2017) | 15 / 582656 / 37645 | Moderate | 1.09 [0.93, 1.28] * | | 0.84 | 0 | 0 | 0 | 0 | 0 | 1 |
|  | Yang (2019) | 20 / 682622 / 42084 | Low | 1.06 [0.96, 1.19] | | 0.81 | 0 | 0 | 1 | 0 | 0 | 0 |
|  | Zhou (2011) | 5 / 202507 / 10020 | Low | 1.04 [1.02, 1.06] | | 0 | 0 | 0 | 0 | 0 | 0 | 0 |
| Fruit | Halvorsen (2020) | 19 / 1496860 / 80994 | High | 0.98 [0.97, 1.00] | | 0.68 | 1 | 1 | 1 | 1 | 1 | 1 |
|  | Li (2014) | 7 / NA / NA | High | 0.95 [0.92, 1.00] | | 0 | 0 | 0 | 0 |  |  | 1 |
|  | Schwingshackl (2017) | 13 / 850985 / 53317 | Moderate | 0.98 [0.98, 1.00] | | 0.21 | 0 | 0 | 0 | 0 | 0 | 0 |
|  | Carter (2009) | 5 / 204654 / 8563 | Low | 0.95 [0.87, 1.04] | | 0.53 | 0 | 0 | 0 | 0 | 0 | 0 |
|  | Wu (2014) | 9 / 391148 / 27998 | Low | 0.99 [0.98, 1.00] | | 0.18 | 0 | 0 | 0 | 0 | 0 | 0 |
| Legumes | Schwingshackl (2017) | 10 / 536860 / 29223 | Moderate | 1.00 [0.92, 1.09] * | | 0.87 | 1 | 0 | 1 | 1 | 1 | 0 |
|  | Afshin (2013) | 2 / 100179 / 2746 | Low | 0.88 [0.71, 1.11] | | 0.96 | 0 | 0 | 0 | 0 | 0 | 0 |
|  | Tang (2019) | 7 / 271709 / 11232 | High | 0.95 [0.80, 1.14] | | NA | 0 | 1 | 0 | 0 | 0 | 1 |
| Nuts | Schwingshackl (2017) | 7 / 297012 / 15470 | Moderate | 0.89 [0.71, 1.12] * | | 0.77 | 1 | 0 | 1 | 0 | 1 | 0 |
|  | Afshin (2013) | 6 / 230216 / 13308 | Low | 0.87 [0.81, 0.94] * | | 0.22 | 0 | 0 | 0 | 0 | 0 | 0 |
|  | Aune (2016) | 4 / 193928 / 800 | High | 0.61 [0.43, 0.88] * | | 0 | 0 | 0 | 0 | 0 | 0 | 1 |
|  | Becerra-Tomas (2020) | 4 / 194168 / 12878 | Low | 1.03 [0.93, 1.13] * | | 0.6 | 0 | 1 | 0 | 0 | 0 | 0 |
|  | Luo (2013) | 2 / 2763915 / 9219 | Moderate | 0.88 [0.84, 0.92] * | | 0 | 0 | 0 | 0 | 1 | 0 | 0 |
|  | Zhou (2013) | 6 / 342213 / 14400 | Low | 0.80 [0.57, 1.14] * | | 0.87 | 0 | 0 | 0 | 0 | 0 | 0 |
| Processed meat | Shi (2022) | 18 / 1187449 / 20146 | Moderate | 1.44 [1.27, 1.63] * | | 0.91 | 1 | 1 | 1 | 1 | 0 | 1 |
|  | Aune (2008) | 8 / 380606 / 9999 | Low | 1.57 [1.28, 1.93] * | | 0.68 | 0 | 0 | 0 | 0 | 0 | 0 |
|  | Fan (2019) | 16 / 512033 / 29954 | Low | 1.41 [1.24, 1.60] * | | 0.86 | 0 | 0 | 0 | 0 | 0 | 0 |
|  | Schwingshackl (2017) | 14 / 550342 / 43781 | Moderate | 1.37 [1.22, 1.55] * | | 0.88 | 0 | 0 | 0 | 0 | 0 | 1 |
|  | Yang (2019) | 17 / 663144 / 49086 | Low | 1.46 [1.26, 1.69] * | | 0.93 | 0 | 0 | 0 | 0 | 1 | 0 |
| Red meat | Shi (2022) | 18 / 1650241 / 20146 | Moderate | 1.27 [1.16, 1.39] * | | 0.89 | 1 | 1 | 1 | 1 | 0 | 1 |
|  | Aune (2008) | 9 / 433070 / 12226 | Low | 1.16 [1.03, 1.31] | | 0.68 | 0 | 0 | 0 | 0 | 0 | 0 |
|  | Fan (2019) | 16 / 657454 / 48400 | Low | 1.23 [1.12, 1.35] | | 0.76 | 0 | 0 | 0 | 0 | 0 | 0 |
|  | Schwingshackl (2017) | 14 / 550342 / 43781 | Moderate | 1.17 [1.08, 1.26] * | | 0.83 | 0 | 0 | 0 | 0 | 0 | 1 |
|  | Yang (2019) | 17 / 663144 / 49086 | Low | 1.31 [1.19, 1.45] * | | 0.76 | 0 | 0 | 0 | 0 | 1 | 0 |
| Refined grains | Schwingshackl (2017) | 14 / 599772 / 22559 | Moderate | 1.01 [0.99, 1.03] * | | 0.59 | 1 | 1 | 1 | 1 | 1 | 1 |
|  | Aune (2013) | 6 / 258078 / 9545 | Low | 0.98 [0.96, 1.01] | | 0.53 | 0 | 0 | 0 | 0 | 0 | 0 |
| SSBs | Qin (2019) | 19 / 1010392 / 34788 | Moderate | 1.19 [1.13, 1.25] * | | 0.82 | 1 | 0 | 1 | 1 | 0 | 0 |
|  | Greenwood (2013) | 6 / 295223 / 26092 | High | 1.15 [1.09, 1.21] | | 0.8 | 0 | 0 | 0 | 0 | 0 | 1 |
|  | Imamura (2014) | 17 / 464937 / 38253 | High | 1.25 [1.14, 1.37] * | | 0.89 | 0 | 0 | 0 | 0 | 1 | 1 |
|  | Li (2022) | 17 / 566178 / 30666 | High | 1.20 [1.15, 1.25] * | | NA | 0 | 1 | 0 | 0 | 0 | 1 |
|  | Meng (2020) | 13 / 445040 / 15768 | High | 1.27 [1.15, 1.41] * | | 0.81 | 0 | 0 | 0 | 0 | 0 | 1 |
|  | Schwingshackl (2017) | 10 / 352937 / 30887 | Moderate | 1.21 [1.12, 1.31] * | | 0.78 | 0 | 0 | 0 | 0 | 0 | 0 |
| Vegetables | Halvorsen (2020) | 15 / 784014 / 52333 | High | 0.98 [0.97, 1.00] | | 0.39 | 1 | 1 | 1 | 1 | 1 | 1 |
|  | Carter (2009) | 5 / 204654 / 8563 | Low | 0.91 [0.77, 1.09] | | 0.78 | 0 | 0 | 0 | 0 | 0 | 0 |
|  | Li (2014) | 5 / 290927 / 20933 | High | 0.98 [0.90, 1.08] | | 0.46 | 0 | 0 | 0 | 0 | 0 | 1 |
|  | Schwingshackl (2017) | 11 / 734949 / 45648 | Moderate | 0.98 [0.96, 1.00] * | | 0.62 | 0 | 0 | 0 | 0 | 0 | 0 |
|  | Wu (2014) | 7 / 275112 / 20329 | Low | 0.98 [0.95, 1.01] * | | 0.78 | 0 | 0 | 0 | 0 | 0 | 0 |
| White meat | Yang (2019) | 11 / 411276 / 32983 | Low | 1.08 [1.00, 1.19] | | 0 | 1 | 0 | 1 | 1 | 1 | 1 |
|  | Fan (2019) | 8 / 219926 / 13865 | Low | 1.04 [0.96, 1.14] | | 0 | 0 | 1 | 0 | 0 | 0 | 1 |
| Whole grains | Ghanbari-Gohari (2021) | 9 / 436282 / 37249 | Moderate | 0.85 [0.80, 0.92] | | 0.94 | 1 | 1 | 0 | 0 | 1 | 1 |
|  | Aune (2013) | 10 / 385868 / 19829 | Low | 0.88 [0.83, 0.93] | | 0.82 | 0 | 0 | 0 | 0 | 0 | 0 |
|  | Schwingshackl (2017) | 12 / 459603 / 22267 | Moderate | 0.87 [0.82, 0.93] * | | 0.91 | 0 | 0 | 1 | 1 | 0 | 1 |
| Incidence of metabolic syndrome | | | | | | | | | | | | |
| Dairy | Kim (2015) | 4 / 31944 / 6870 | Low | 0.88 [0.82, 0.95] * | | 0.55 | 1 | 0 | 0 | 1 | 1 | 1 |
|  | Chen (2014) | 5 / 19897 / NA | Low | 0.94 [0.90, 0.98] * | | NA | 0 | 0 | 1 | 0 | NA | 1 |
|  | Lee (2016) | 3 / 9259 / 2227 | Low | 0.88 [0.80, 0.97] * | | 0.44 | 0 | 1 | 0 | 0 | 0 | 1 |
| Fish | Karimi (2019) | 5 / 23647 / NA | High | 0.80 [0.66, 0.96] * | | 0.5 | 1 | 1 | 1 | 1 | NA | 1 |
|  | Kim (2014) | 2 / 7860 / 1671 | Low | 0.65 [0.48, 0.87] | | 0.66 | 0 | 0 | 0 | 0 | 1 | 0 |
| Fruit | Lee (2018) | 2 / 8867 / NA | High | 1.00 [0.97, 1.02] | | 0 | 1 | 1 | 1 | 1 | NA | 1 |
| Nuts | Li (2017) | 3 / 20666 / 4625 | High | 0.96 [0.93, 0.99] | | 0 | 1 | 1 | 1 | 1 | 1 | 1 |
| SSBs | Zhang (2014) | 5 / 27238 / 7986 | Moderate | 1.15 [0.97, 1.36] * | | 0.79 | 1 | 1 | 1 | 1 | 1 | 1 |
| Vegetables | Lee (2018) | 2 / 8867 / NA | High | 0.76 [0.27, 1.26] * | | 0.54 | 1 | 1 | 1 | 1 | NA | 1 |

SSBs: sugar-sweetened beverages. *The result is converted to these serving sizes for each food group (g/day): dairy: 200; eggs: 50; fish: 100; fruits: 80; legumes: 50; nuts: 28; processed meat: 50; red meat: 100; refined grains: 30 (product weight/fresh weight); sugar-sweetened beverages (SSBs): 250 or 250 mL/day; vegetables: 100; white meat: 100; whole grains: 30 (product weight/fresh weight).

# Supplementary Table 6: Table of extracted data for dose-response comparisons categorized per food for associations between food groups and incidence of diabetes, diabetes mortality, and incidence of metabolic syndrome.

| **Food_group** | **Author (Year)** | **Numbers** | **AMSTAR-2** | **Range** |
| --- | --- | --- | --- | --- |
| Dairy | Aune (2013) | N. of studies: 12 - N. of participants: 426055 - N. of events: 26976 | Low | [0 - 700] |
|  | Fan (2019) | N. of studies: 7 - N. of participants: 98606 - N. of events: 5662 | Low | [0 - 700] |
|  | Feng (2021) | N. of studies: 20 - N. of participants: 560852 - N. of events: 36291 | High | [0 - 1600] |
|  | Gijsbers (2015) | N. of studies: 16 - N. of participants: 489113 - N. of events: 38993 | Low | [0 - 700] |
|  | Schwingshackl (2017) | N. of studies: 21 - N. of participants: 566872 - N. of events: 44474 | Moderate | [0 - 2000] |
|  | Chen (2014) | N. of studies: 5 - N. of participants: 19897 - N. of events: NA | Low | [0 - 6] |
| Eggs | Fan (2019) | N. of studies: 19 - N. of participants: 374891 - N. of events: 20691 | Low | [0 - 60] |
|  | Schwingshackl (2017) | N. of studies: 13 - N. of participants: 315358 - N. of events: 17629 | Moderate | [0 - 60] |
| Fish | Fan (2019) | N. of studies: 8 - N. of participants: 698894 - N. of events: 43239 | Low | [0 - 160] |
|  | Schwingshackl (2017) | N. of studies: 15 - N. of participants: 582656 - N. of events: 37645 | Moderate | [0 - 225] |
|  | Yang (2019) | N. of studies: 20 - N. of participants: 682622 - N. of events: 42084 | Low | [0 - 175] |
|  | Zhou (2011) | N. of studies: 5 - N. of participants: 202507 - N. of events: 10020 | Low | [0 - 420] |
|  | Karimi (2019) | N. of studies: 5 - N. of participants: 23647 - N. of events: NA | High | [0 - 800] |
| Fruit | Halvorsen (2020) | N. of studies: 19 - N. of participants: 1496860 - N. of events: 80994 | High | [0 - 600] |
|  | Li (2014) | N. of studies: 7 - N. of participants: NA - N. of events: NA | High | [0 - 5] |
|  | Schwingshackl (2017) | N. of studies: 13 - N. of participants: 850985 - N. of events: 53317 | Moderate | [0 - 618] |
|  | Wu (2014) | N. of studies: 9 - N. of participants: 391148 - N. of events: 27998 | Low | [0 - 4] |
|  | Lee (2018) | N. of studies: 2 - N. of participants: 8867 - N. of events: NA | High | [0 - 550] |
| Legumes | Schwingshackl (2017) | N. of studies: 10 - N. of participants: 536860 - N. of events: 29223 | Moderate | [0 - 190] |
|  | Tang (2019) | N. of studies: 7 - N. of participants: 271709 - N. of events: 11232 | High | [0 - 100] |
| Nuts | Aune (2016) | N. of studies: 4 - N. of participants: 193928 - N. of events: 800 | High | [0 - 25] |
|  | Schwingshackl (2017) | N. of studies: 7 - N. of participants: 297012 - N. of events: 15470 | Moderate | [0 - 30] |
| Processed meat | Fan (2019) | N. of studies: 16 - N. of participants: 512033 - N. of events: 29954 | Low | [0 - 150] |
|  | Schwingshackl (2017) | N. of studies: 14 - N. of participants: 550342 - N. of events: 43781 | Moderate | [0 - 142] |
|  | Yang (2019) | N. of studies: 17 - N. of participants: 663144 - N. of events: 49086 | Low | [0 - 140] |
| Red meat | Fan (2019) | N. of studies: 16 - N. of participants: 657454 - N. of events: 48400 | Low | [0 - 180] |
|  | Schwingshackl (2017) | N. of studies: 14 - N. of participants: 550342 - N. of events: 43781 | Moderate | [0 - 207] |
|  | Yang (2019) | N. of studies: 17 - N. of participants: 663144 - N. of events: 49086 | Low | [0 - 140] |
| Refined grains | Aune (2013) | N. of studies: 6 - N. of participants: 258078 - N. of events: 9545 | Low | [0 - 7] |
|  | Schwingshackl (2017) | N. of studies: 14 - N. of participants: 599772 - N. of events: 22559 | Moderate | [0 - 700] |
| SSBs | Greenwood (2013) | N. of studies: 6 - N. of participants: 295223 - N. of events: 26092 | High | [0 - 1000] |
|  | Meng (2020) | N. of studies: 13 - N. of participants: 445040 - N. of events: 15768 | High | [0 - 20] |
|  | Qin (2019) | N. of studies: 19 - N. of participants: 1010392 - N. of events: 34788 | Moderate | [0 - 1800] |
|  | Schwingshackl (2017) | N. of studies: 10 - N. of participants: 352937 - N. of events: 30887 | Moderate | [0 - 748] |
|  | Zhang (2014) | N. of studies: 5 - N. of participants: 27238 - N. of events: 7986 | Moderate | [0 - 1100] |
| Vegetables | Halvorsen (2020) | N. of studies: 15 - N. of participants: 784014 - N. of events: 52333 | High | [0 - 700] |
|  | Schwingshackl (2017) | N. of studies: 11 - N. of participants: 734949 - N. of events: 45648 | Moderate | [0 - 636] |
|  | Wu (2014) | N. of studies: 7 - N. of participants: 275112 - N. of events: 20329 | Low | [0 - 7] |
| White meat | Fan (2019) | N. of studies: 8 - N. of participants: 219926 - N. of events: 13865 | Low | [0 - 100] |
|  | Yang (2019) | N. of studies: 11 - N. of participants: 411276 - N. of events: 32983 | Low | [0 - 80] |
| Whole grains | Aune (2016) | N. of studies: 4 - N. of participants: 632849 - N. of events: 808 | Moderate | [0 - 250] |
|  | Aune (2013) | N. of studies: 10 - N. of participants: 385868 - N. of events: 19829 | Low | [0 - 5] |
|  | Ghanbari-Gohari (2021) | N. of studies: 9 - N. of participants: 436282 - N. of events: 37249 | Moderate | [0 - 150] |
|  | Schwingshackl (2017) | N. of studies: 12 - N. of participants: 459603 - N. of events: 22267 | Moderate | [0 - 100] |

SSBs: sugar-sweetened beverages.

# Supplementary Table 7: Overview of the most comprehensive/up-to-date meta-analyses on associations between food groups and diabetes-related mortality with details on first author, search year, study quality, exposure, number of studies, participants, events, result and certainty of evidence.

| Food group | Author (year) | Study quality* | Comparison | Studies/participants/events | Results | Certainty of evidence** |
| --- | --- | --- | --- | --- | --- | --- |
| Dairy | Barbaresko (2022) | High | PS (200g) | 4 / 14493 / 1913 | 1.00 [0.96, 1.08] (I^2^ = 0.00) | Low |
| Eggs | Barbaresko (2022) | High | PS (50g) | 7 / 64545 / 3087 | 1.28 [1.10, 1.40] (I^2^ = 0.56) | Low |
| Fish | Jayedi (2019) | High | HL | 8 / 57077 / NA | 0.86 [0.76, 0.96] (I^2^ = 0.50) | Moderate |
| Fish | Barbaresko (2022) | High | PS (100g) | 6 / 24854 / 3835 | 0.95 [0.92, 0.99] (I^2^ = 0.00) | Low |
| Fruit | Barbaresko (2022) | High | PS (80g) | 4 / 65164 / 6613 | 0.85 [0.78, 0.94] (I^2^ = 0.68) | Low |
| Meat | Barbaresko (2022) | High | PS (50 g) | 3 / 13610 / 1426 | 1.03 [0.91, 1.15] (I^2^ = 0.00) | Low |
| Nuts | Barbaresko (2022) | High | PS (28g) | 2 / 22601 / 6512 | 0.32 [0.10, 1.32] (I^2^ = 0.84) | Low |
| Vegetables | Barbaresko (2022) | High | PS (100g) | 2 / 16662 / 1862 | 0.88 [0.82, 0.94] (I^2^ = 0.00) | Low |
| Whole grains | Aune (2016) | Moderate | HL | 4 / 632849 / 808 | 0.64 [0.42, 0.98] (I^2^ = 0.64) | Moderate |
| Whole grains | Barbaresko (2022) | High | PS (30g) | 2 / 14035 / 1368 | 0.77 [0.60, 0.99] (I^2^ = 0.00) | Low |

HL: high versus low. PS: Per serving with serving sizes in the parentheses. *AMSTAR2 score. **NutriGrade scoring system for meta-analysis of cohort studies.

# **Supplementary Table 8**: Overview of the most comprehensive/up-to-date meta-analyses on associations between food groups and incidence of metabolic syndrome with details on first author, search year, study quality, exposure, number of studies, participants, events, results and certainty of evidence.

| Food group | Author (year) | Study quality* | Comparison | Studies/participants/events | Results | Certainty of evidence** |
| --- | --- | --- | --- | --- | --- | --- |
| Dairy | Mena-Sanchez (2017) | Low | HL | 9 / 33001 / 9953 | 0.73 [0.64, 0.83] (I^2^ = 0.62) | Moderate |
| Dairy | Kim (2015) | Low | PS (200g) | 4 / 31944 / 6870 | 0.88 [0.82, 0.95] (I^2^ = 0.55) | Low |
| Eggs | Ding (2021) | High | HL | 4 / 15824 / NA | 0.99 [0.77, 1.26] (I^2^ = 0.53) | Very low |
| Fish | Kim (2014) | Low | HL | 2 / 7860 / 1671 | 0.71 [0.58, 0.87] (I^2^ = 0.61) | Low |
| Fish | Karimi (2019) | High | PS (100g) | 5 / 23647 / NA | 0.80 [0.66, 0.96] (I^2^ = 0.50) | Low |
| Fruit | Zhang (2017) | High | HL | 2 / 7379 / NA | 0.81 [0.75, 0.88] (I^2^ = 0.62) | Low |
| Fruit | Lee (2018) | High | PS (80g) | 2 / 8867 / NA | 1.00 [0.97, 1.02] (I^2^ = 0.00) | Low |
| Legumes | Jiang (2019) | Moderate | HL | 2 / 5643 / 1570 | 0.93 [0.77, 1.14] (I^2^ = 0.74) | Low |
| Nuts | Zhang (2018) | Low | HL | 5 / 27304 / NA | 0.84 [0.76, 0.92] (I^2^ = 0.80) | Low |
| Nuts | Li (2017) | High | PS (28g) | 3 / 20666 / 4625 | 0.96 [0.93, 0.99] (I^2^ = 0.00) | Low |
| Processed meat | Guo (2021) | Low | HL | 4 / 5959 / NA | 1.48 [1.11, 1.97] (I^2^ = 0.64) | Low |
| Red meat | Guo (2021) | Low | HL | 3 / 5535 / NA | 1.32 [1.14, 1.54] (I^2^ = 0.54) | Low |
| Refined grains | Guo (2021) | High | HL | 7 / 23985 / NA | 1.10 [0.86, 1.40] (I^2^ = 0.75) | Low |
| SSBs | Muñoz-Cabrejas (2022) | Moderate | HL | 5 / 28932 / 7667 | 1.18 [1.06, 1.32] (I^2^ = 0.70) | Low |
| SSBs | Zhang (2014) | Moderate | PS (250g) | 5 / 27238 / 7986 | 1.15 [0.97, 1.36] (I^2^ = 0.79) | Moderate |
| Vegetables | Zhang (2017) | High | HL | 3 / 8267 / NA | 0.89 [0.85, 0.93] (I^2^ = 0.30) | Low |
| Vegetables | Lee (2018) | High | PS (100g) | 2 / 8867 / NA | 0.76 [0.27, 1.26] (I^2^ = 0.54) | Moderate |
| White meat | Guo (2021) | Low | HL | 3 / 7270 / NA | 0.85 [0.75, 0.97] (I^2^ = 0.00) | Low |
| Whole grains | Guo (2021) | High | HL | 4 / 24115 / NA | 0.91 [0.74, 1.12] (I^2^ = 0.65) | Low |

HL: high versus low. PS: Per serving with serving sizes in the parentheses. SSBs: sugar-sweetened beverages. *AMSTAR2 score. **NutriGrade scoring system for meta-analysis of cohort studies.

# Supplementary Figures 1- 26: Figures presenting estimates for studies for each of the food groups and incidence of diabetes type 2

## Supplementary Figures S1: Associations between intake of whole grains for high versus low and incidence of diabetes type 2 from all meta-analyses


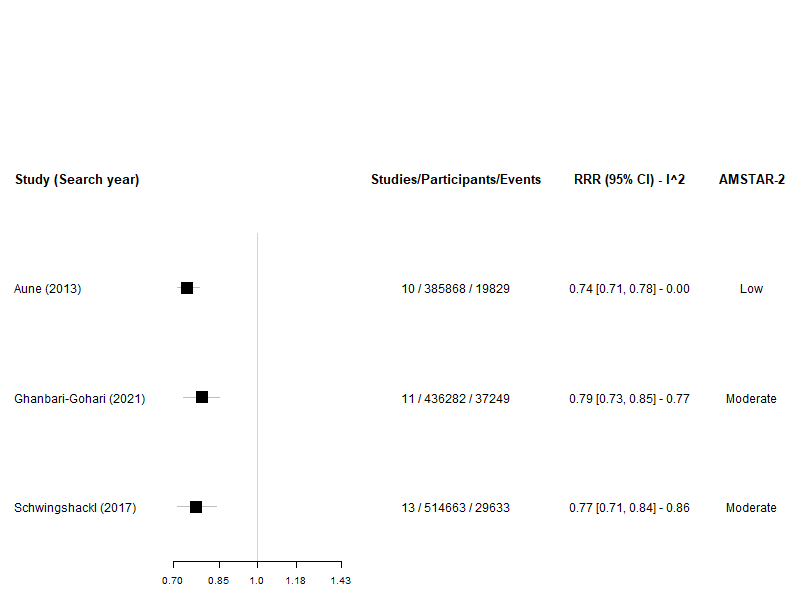


## Supplementary Figures S2: Associations between intake of whole grains per serving and incidence of diabetes type 2 from all meta-analyses


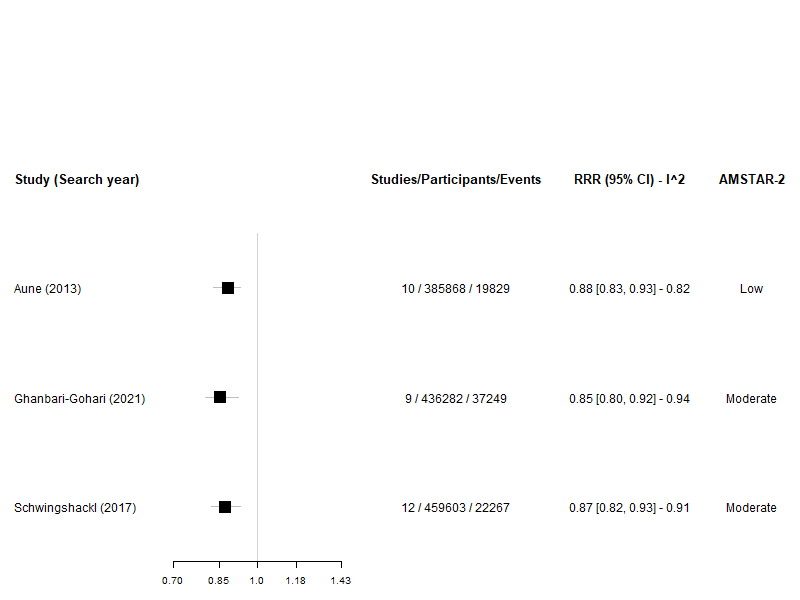


## Supplementary Figures S3: Associations between intake of refined grains for high versus low and incidence of diabetes type 2 from all meta-analyses


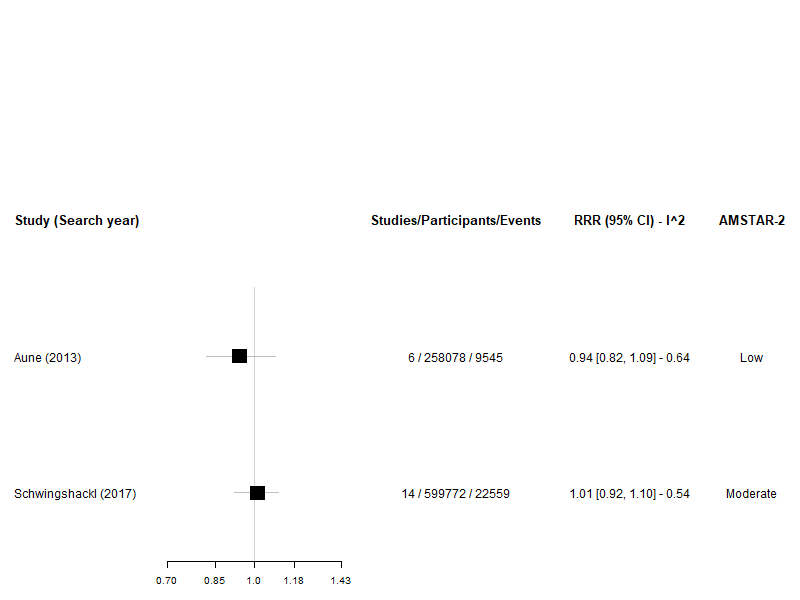


## Supplementary Figures S4: Associations between intake of refined grains per serving and incidence of diabetes type 2 from all meta-analyses


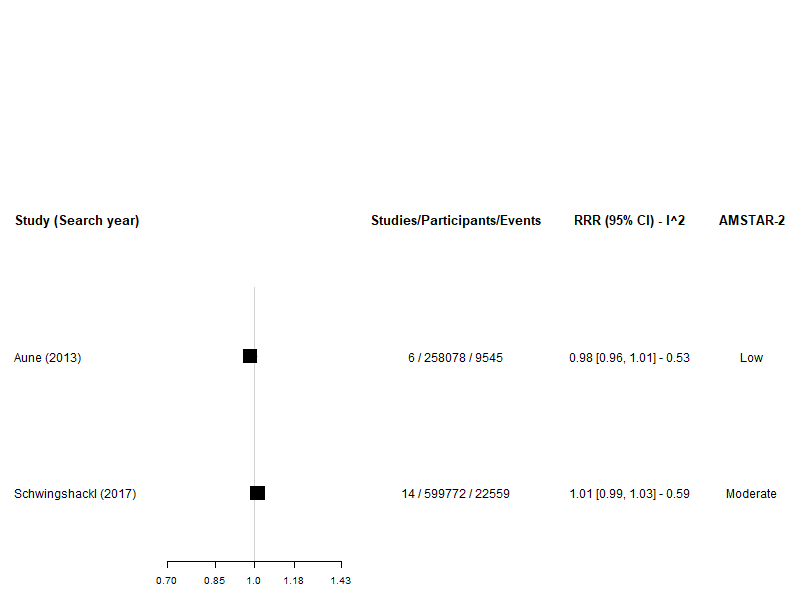


## Supplementary Figures S5: Associations between intake of fruits for high versus low and incidence of diabetes type 2 from all meta-analyses


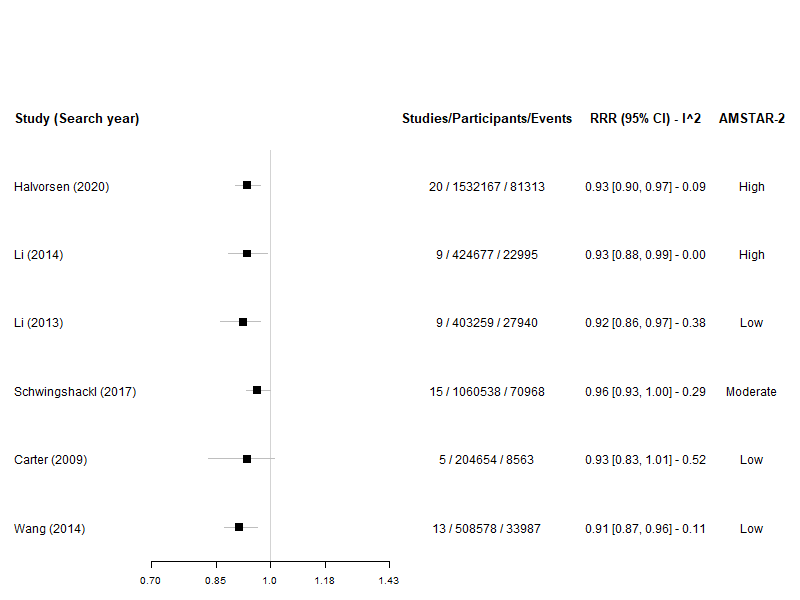


## Supplementary Figures S6: Associations between intake of fruits per serving and incidence of diabetes type 2 from all meta-analyses


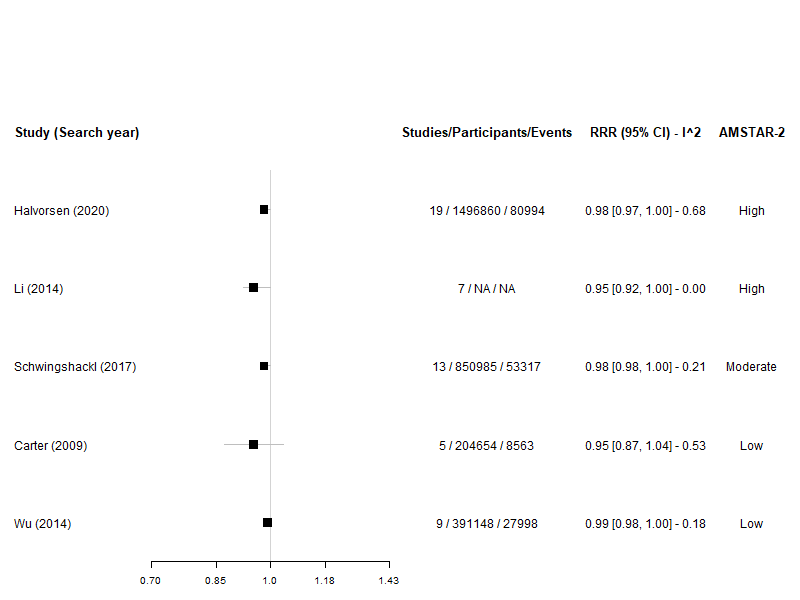


## Supplementary Figures S7: Associations between intake of vegetables for high versus low and incidence of diabetes type 2 from all meta-analyses


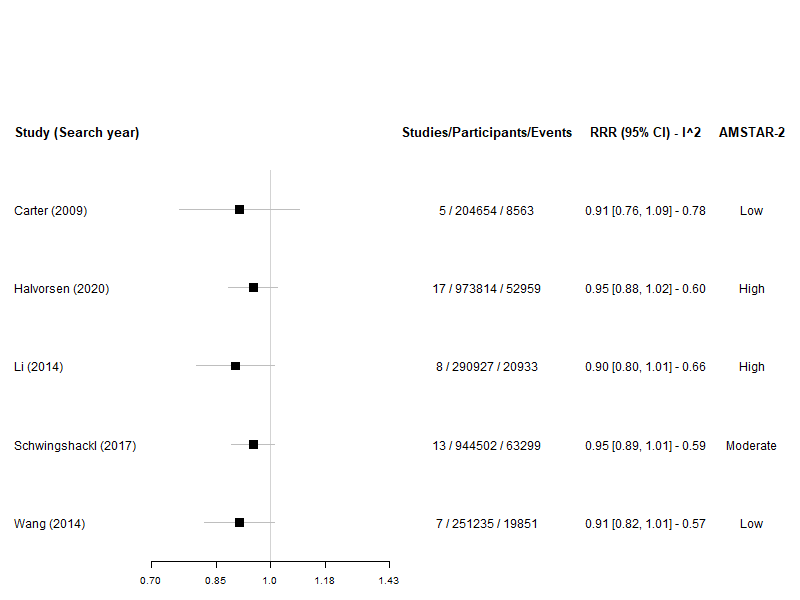


## Supplementary Figures S8: Associations between intake of vegetables per serving and incidence of diabetes type 2 from all meta-analyses


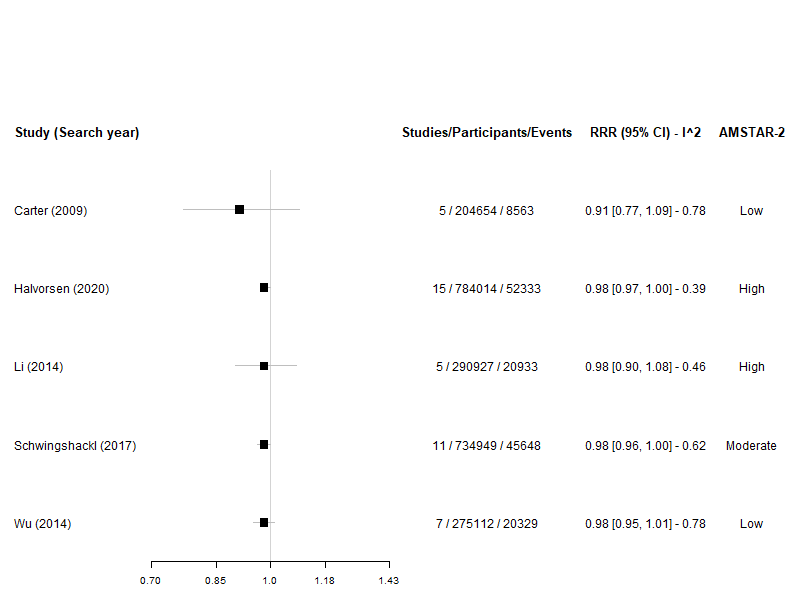


## Supplementary Figures S9: Associations between intake of nuts for high versus low and incidence of diabetes type 2 from all meta-analyses


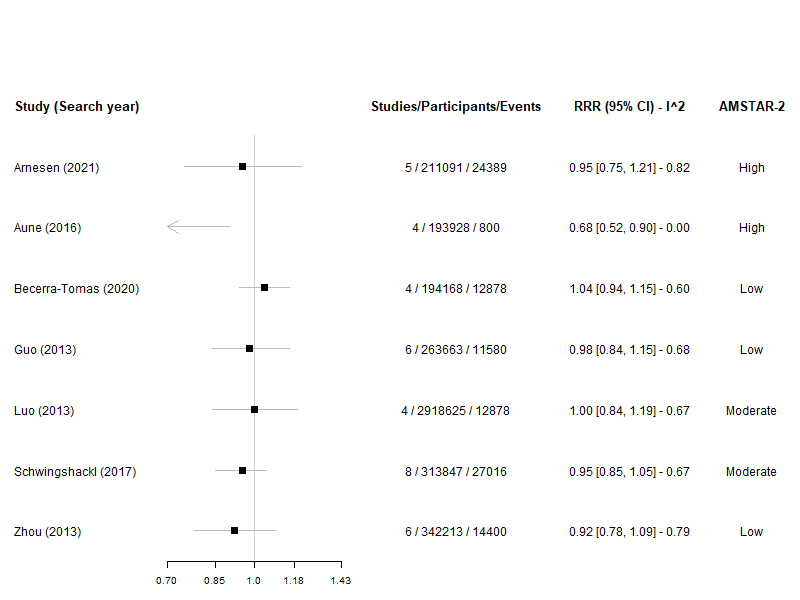


## Supplementary Figures S10: Associations between intake of nuts per serving and incidence of diabetes type 2 from all meta-analyses


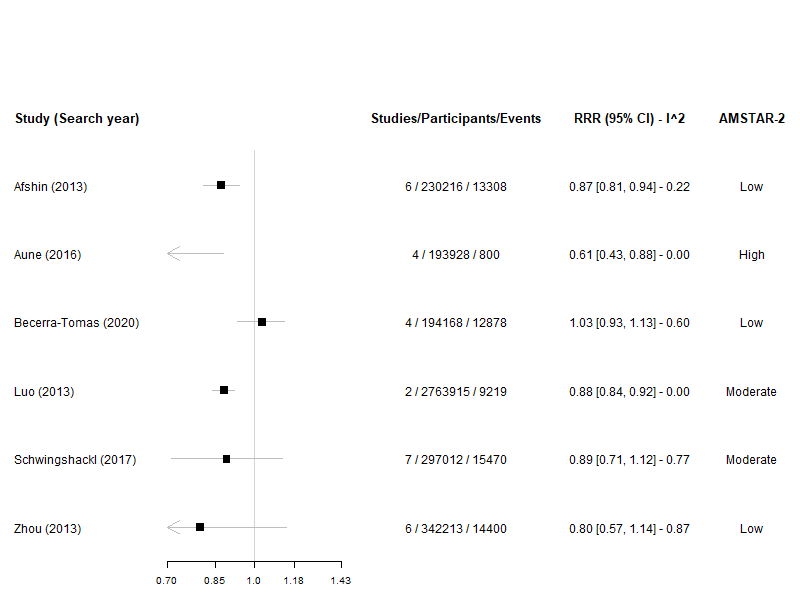


## Supplementary Figures S11: Associations between intake of legumes for high versus low and incidence of diabetes type 2 from all meta-analyses


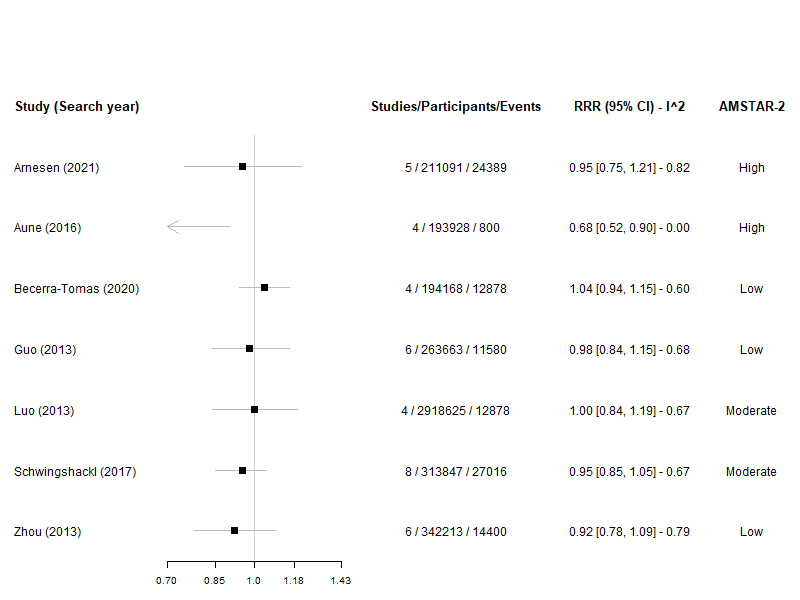


## Supplementary Figures S12: Associations between intake of legumes per serving and incidence of diabetes type 2 from all meta-analyses


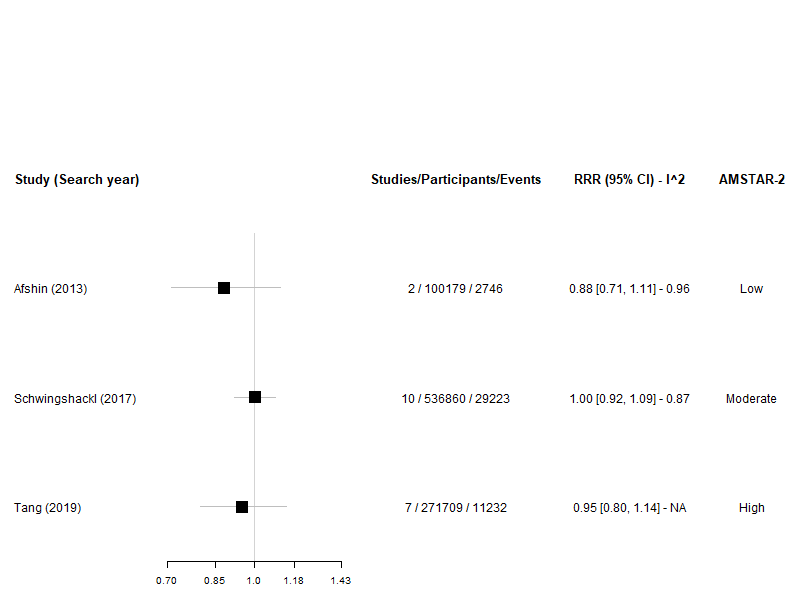


## Supplementary Figures S13: Associations between intake of fish and fish products for high versus low and incidence of diabetes type 2 from all meta-analyses


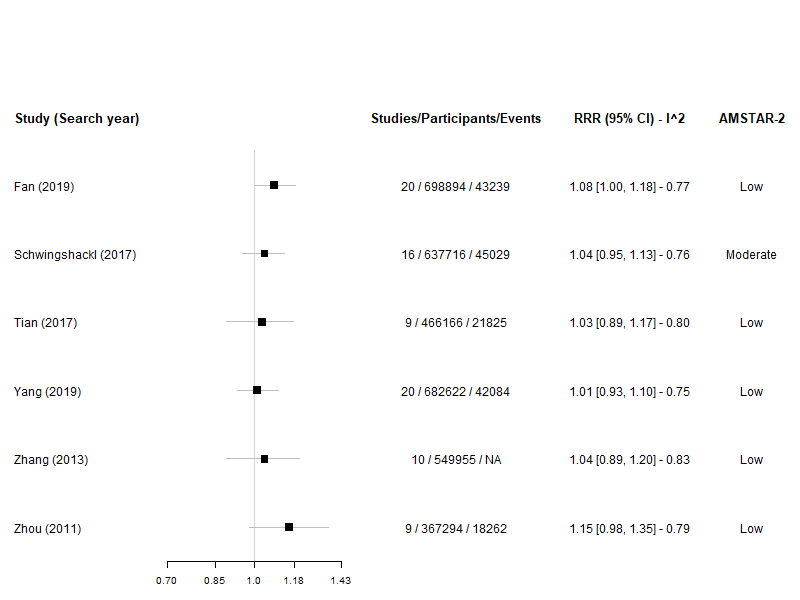


## Supplementary Figures S14: Associations between intake of fish and fish products per serving and incidence of diabetes type 2 from all meta-analyses


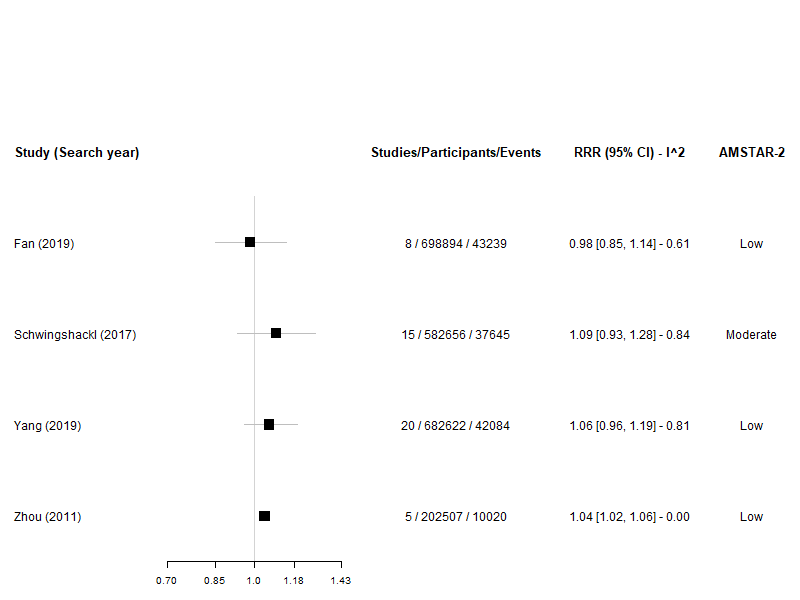


## Supplementary Figures S15: Associations between intake of eggs for high versus low and incidence of diabetes type 2 from all meta-analyses


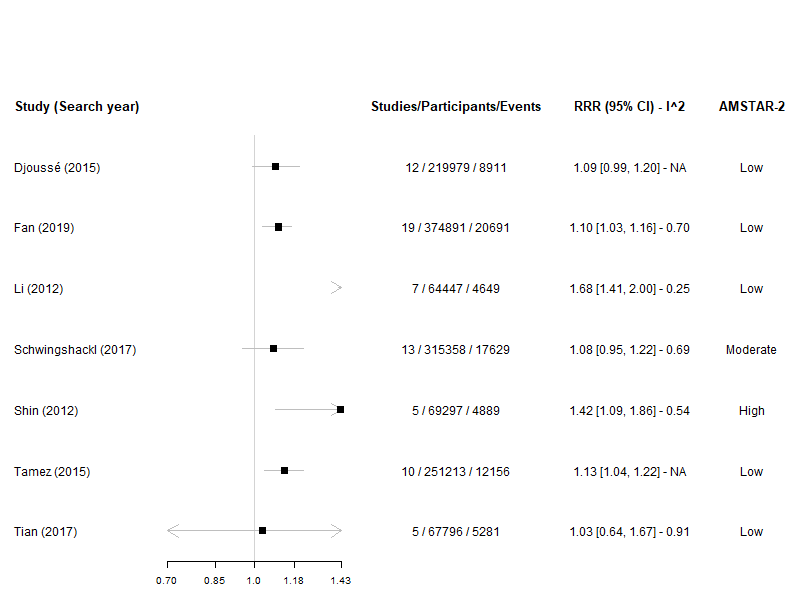


## Supplementary Figures S16: Associations between intake of eggs per serving and incidence of diabetes type 2 from all meta-analyses


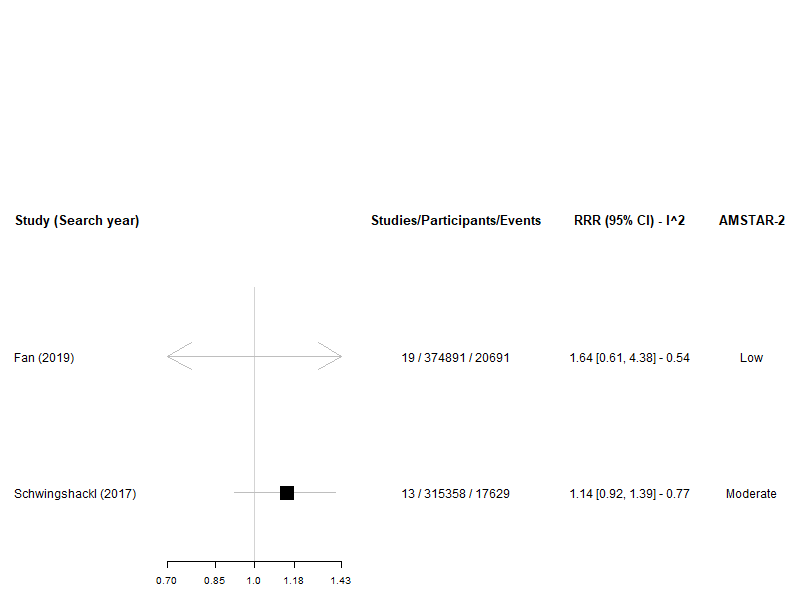


## Supplementary Figures S17: Associations between intake of dairy products for high versus low and incidence of diabetes type 2 from all meta-analyses


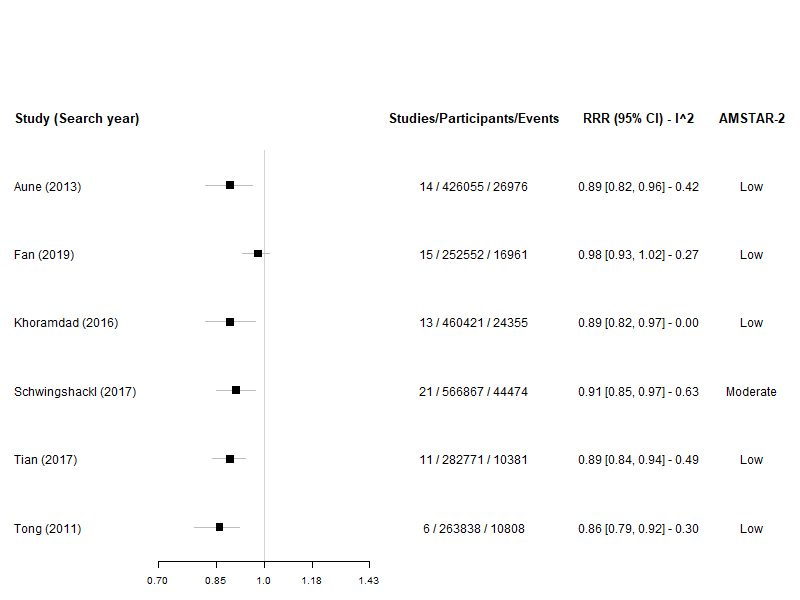


## Supplementary Figures S18: Associations between intake of dairy products per serving and incidence of diabetes type 2 from all meta-analyses


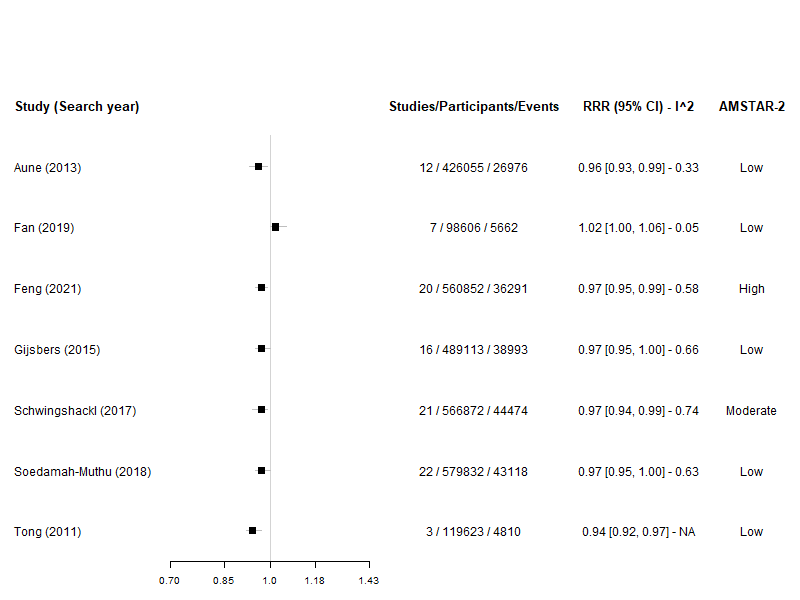


## Supplementary Figures S19: Associations between intake of processed meat for high versus low and incidence of diabetes type 2 from all meta-analyses


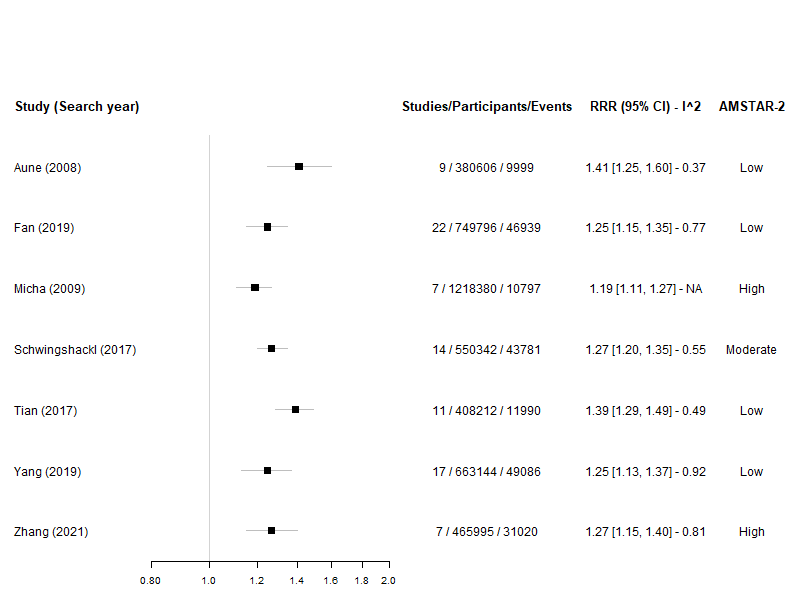


## Supplementary Figures S20: Associations between intake of processed meat per serving and incidence of diabetes type 2 from all meta-analyses


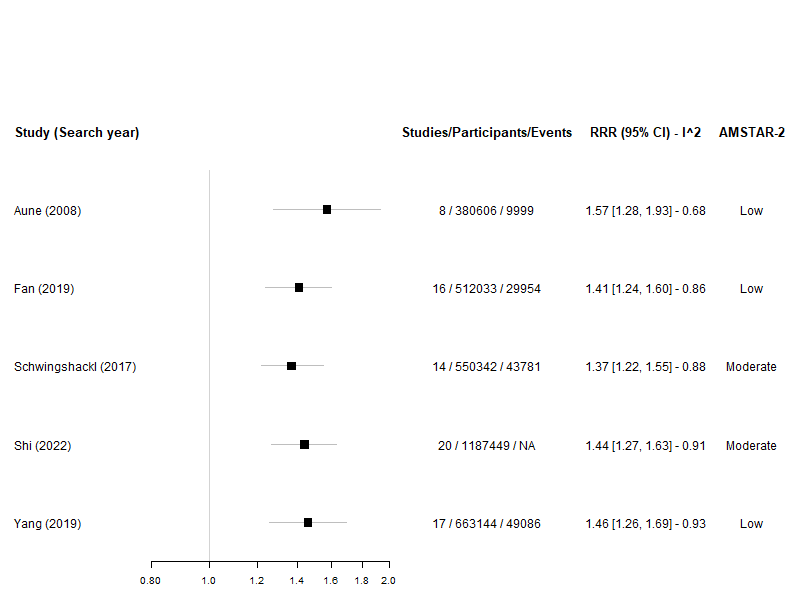


**Supplementary Figures S21: Associations between intake of red meat for high versus low and incidence of diabetes type 2 from all meta-analyses**
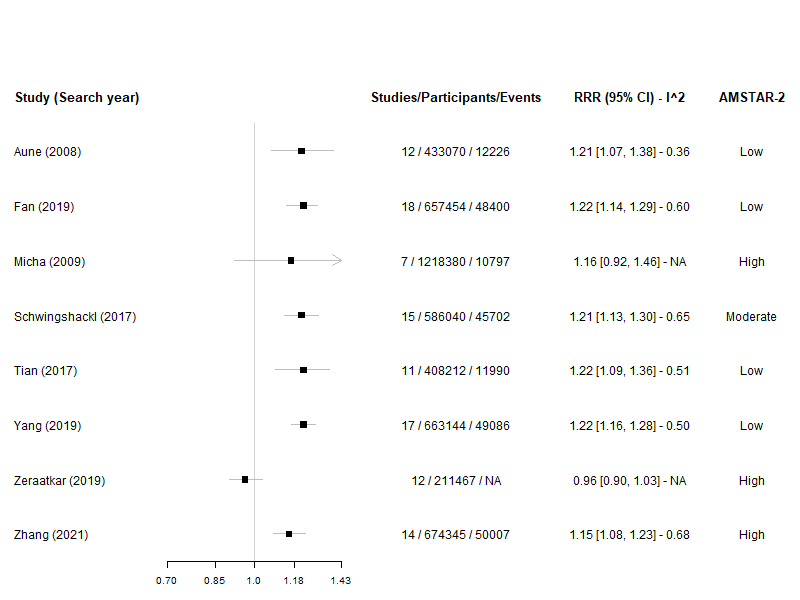


## Supplementary Figures S22: Associations between intake of red meat per serving and incidence of diabetes type 2 from all meta-analyses


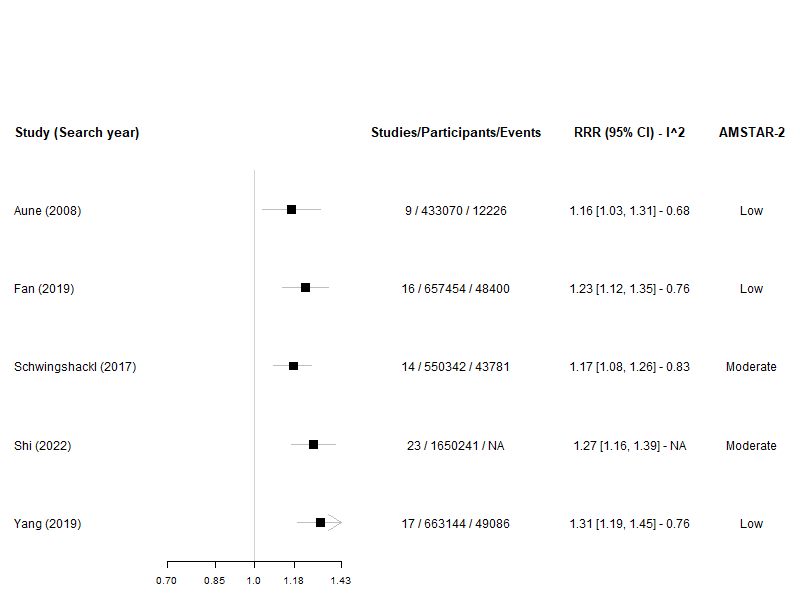


## Supplementary Figures S23: Associations between intake of white meat for high versus low and incidence of diabetes type 2 from all meta-analyses


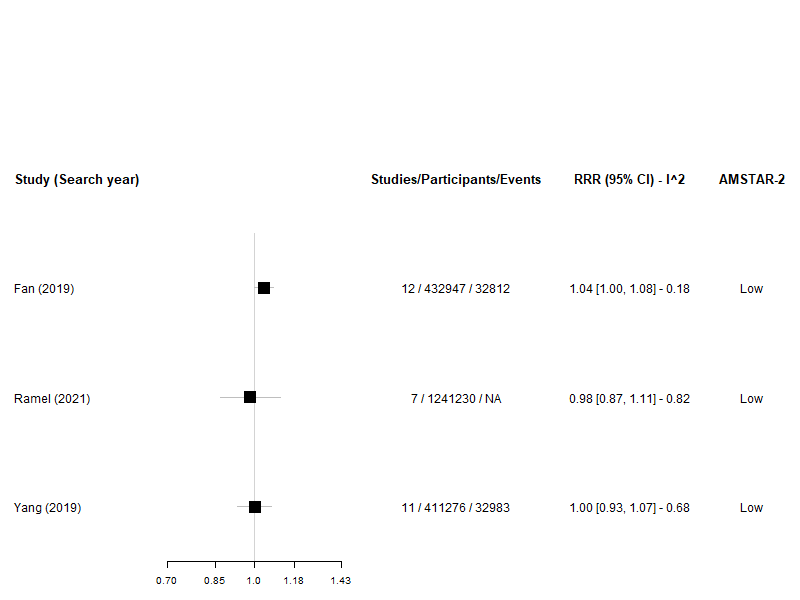


## Supplementary Figures S24: Associations between intake of white meat per serving and incidence of diabetes type 2 from all meta-analyses


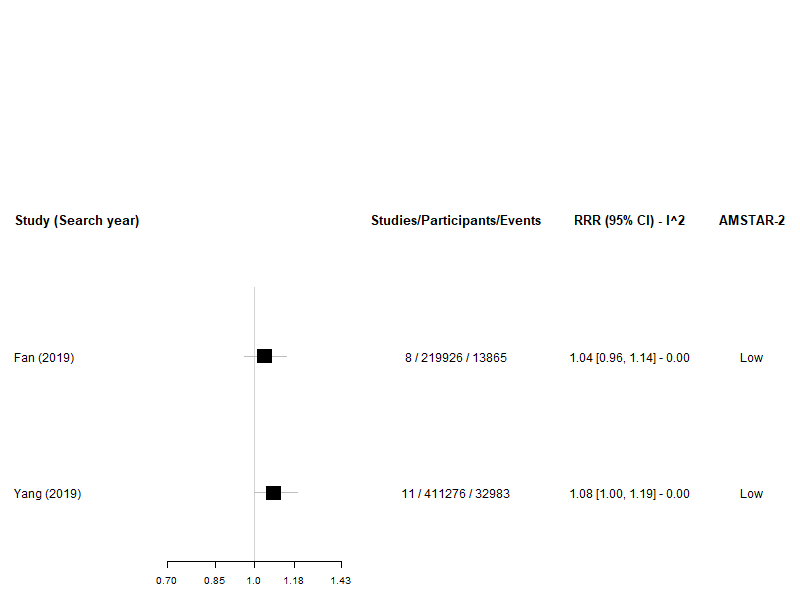


## Supplementary Figures S25: Associations between intake of sugar-sweetened beverages for high versus low and incidence of diabetes type 2 from all meta-analyses


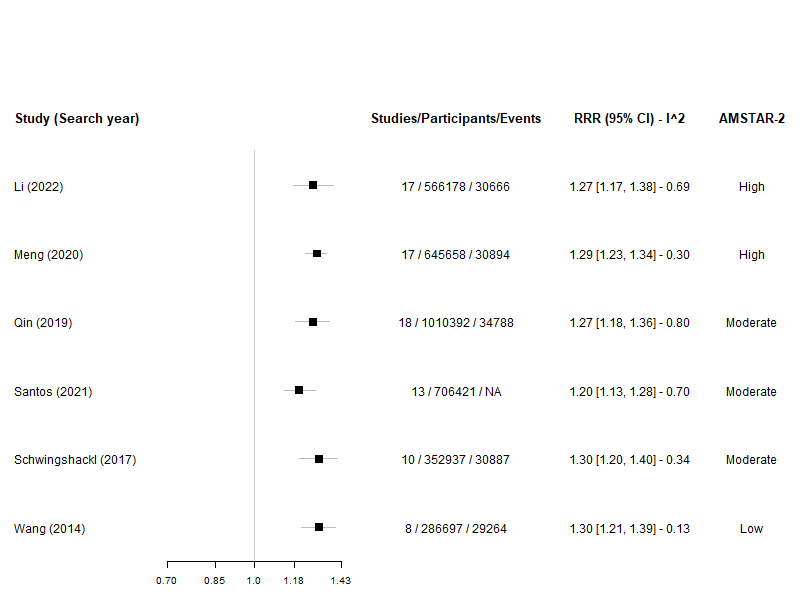


## Supplementary Figures S26: Associations between intake of sugar-sweetened beverages per serving and incidence of diabetes type 2 from all meta-analyses


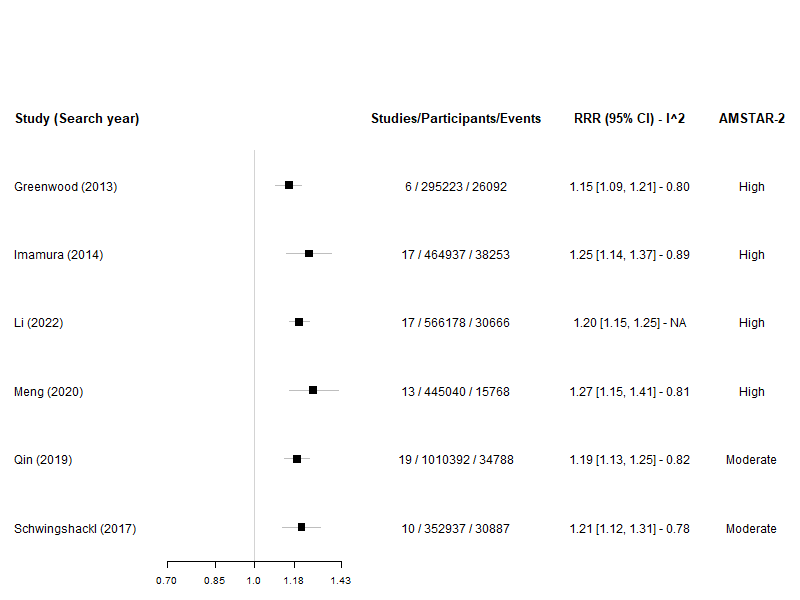


# Supplementary Figure 27: Associations between food groups (high vs. low consumption) and diabetes type 2 mortality in most comprehensive and up-to-date meta-analyses


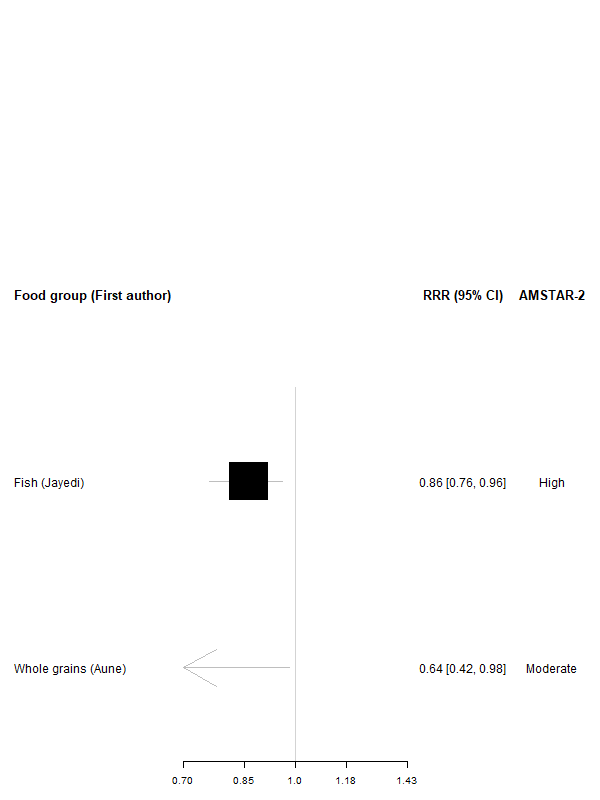


# Supplementary Figure 28: Associations between food groups (per serving) and diabetes type 2 mortality in most comprehensive and up-to-date meta-analyses


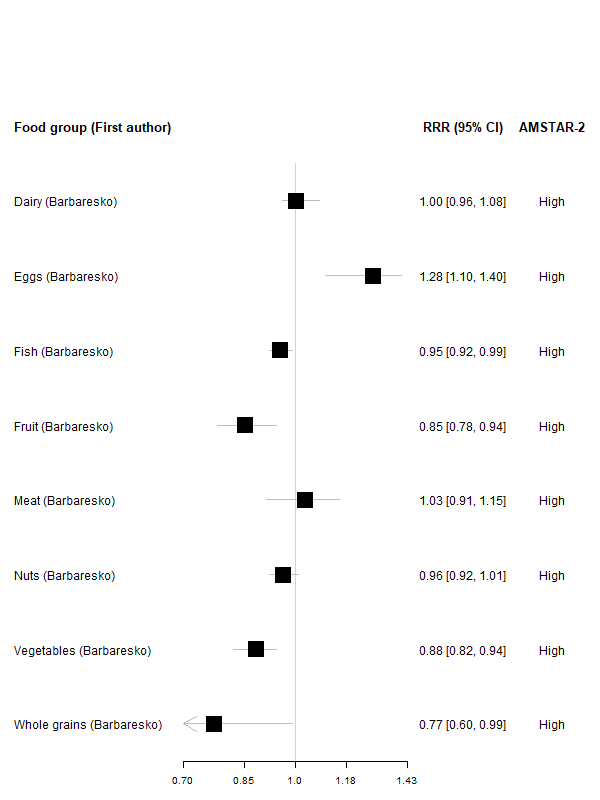


# Supplementary Figures S29: Associations between intake of whole grains per serving and mortality of diabetes type 2 from all meta-analyses


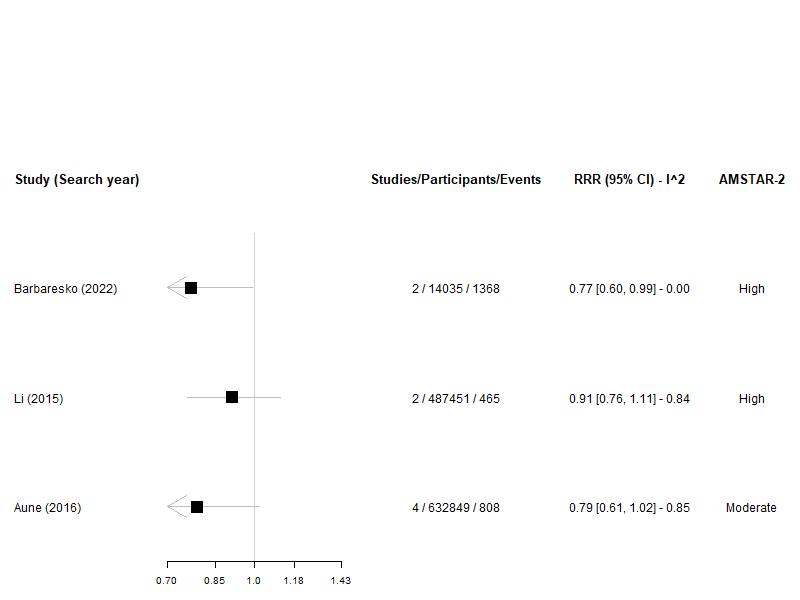


# Supplementary Figure 30: Associations between food groups (high vs. low consumption) and metabolic syndrome incidence in most comprehensive and up-to-date meta-analyses

**
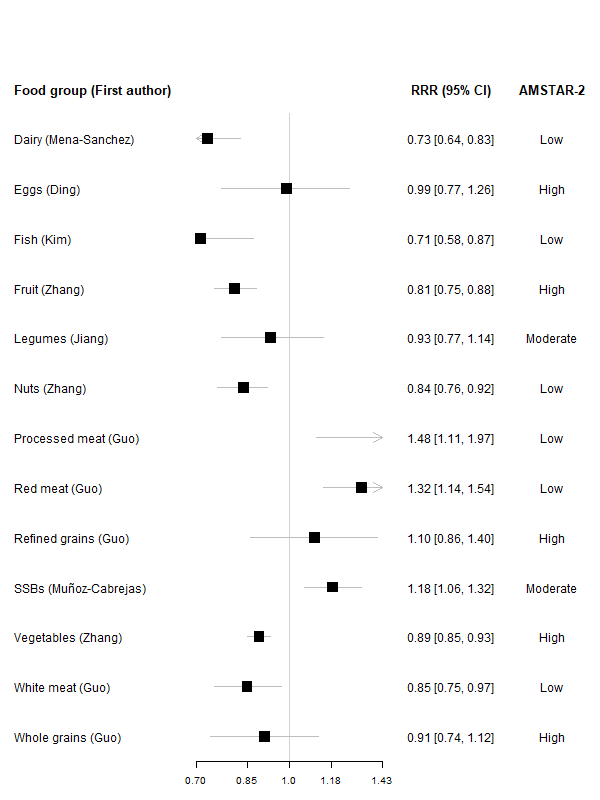
**

# Supplementary Figure 31: Associations between food groups (per serving) and metabolic syndrome incidence in most comprehensive and up-to-date meta-analyses


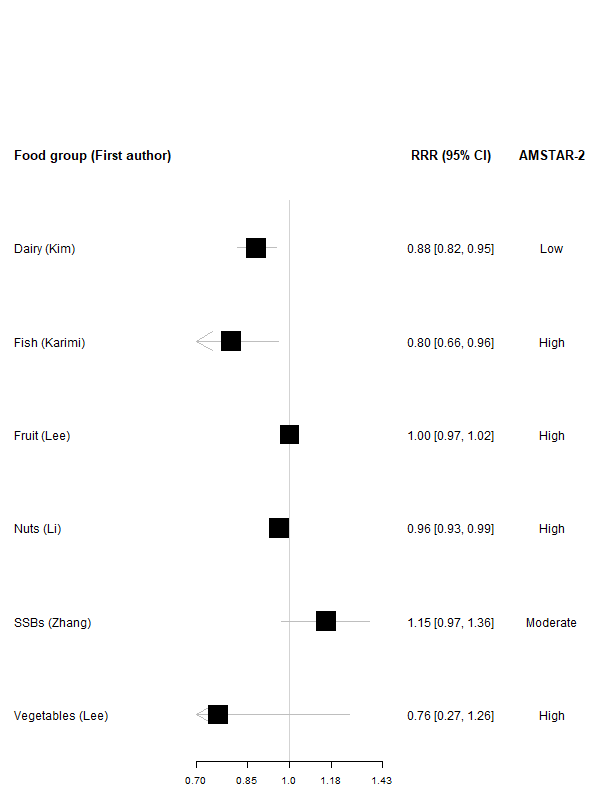


# Supplementary Figures S32: Associations between intake of dairy products for high versus low and incidence of metabolic syndrome from all meta-analyses


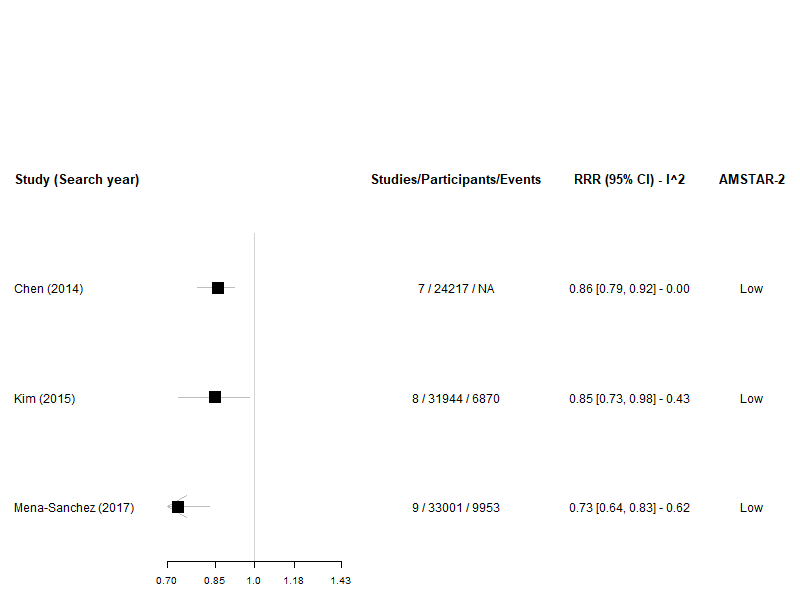


# Supplementary Figures S33: Associations between intake of dairy products per serving and incidence of metabolic syndrome from all meta-analyses


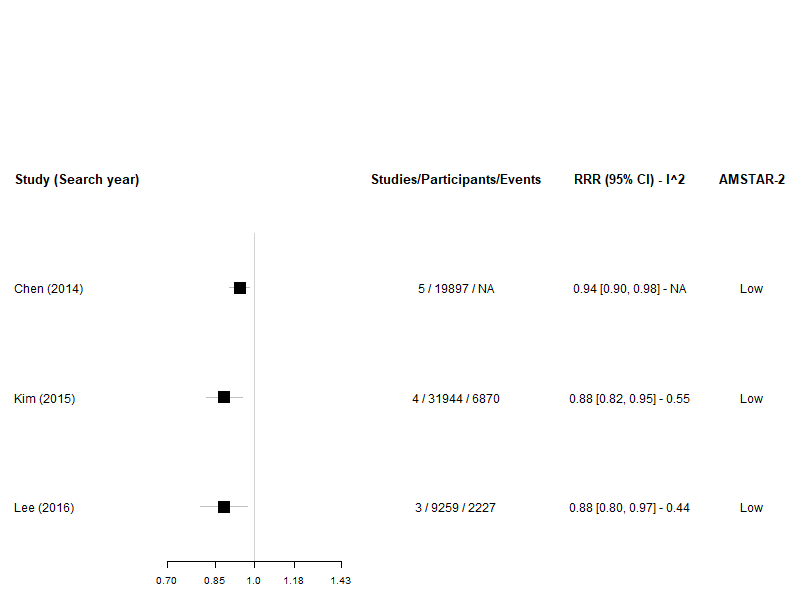


# Supplementary Figures S34: Associations between intake of sugar-sweetened beverages for high versus low and incidence of metabolic syndrome from all meta-analyses


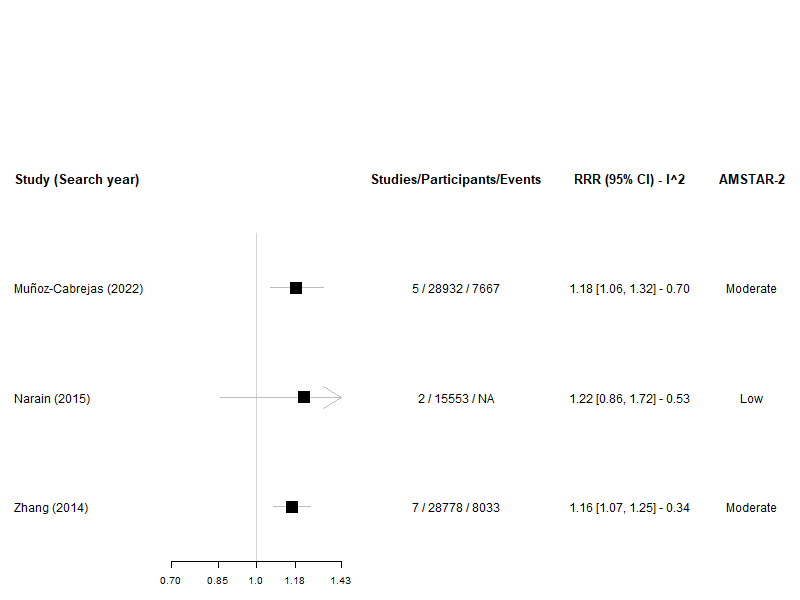

Supplement: multimedia component 2 [file mmc2.docx]
